# Treatment Response, Tumor Infiltrating Lymphocytes and Clinical Outcomes in Inflammatory Breast Cancer–Treated with Neoadjuvant Systemic Therapy

**DOI:** 10.1158/2767-9764.CRC-23-0285

**Published:** 2024-01-24

**Authors:** Maxim De Schepper, Ha-Linh Nguyen, François Richard, Louise Rosias, Florence Lerebours, Roman Vion, Florian Clatot, Anca Berghian, Marion Maetens, Sophia Leduc, Edoardo Isnaldi, Chiara Molinelli, Matteo Lambertini, Federica Grillo, Gabriele Zoppoli, Luc Dirix, Kevin Punie, Hans Wildiers, Ann Smeets, Ines Nevelsteen, Patrick Neven, Anne Vincent-Salomon, Denis Larsimont, Caroline Duhem, Patrice Viens, François Bertucci, Elia Biganzoli, Peter Vermeulen, Giuseppe Floris, Christine Desmedt

**Affiliations:** 1 Laboratory for Translational Breast Cancer Research, Department of Oncology, KU Leuven, Leuven, Belgium.; 2 Department of Pathology, University Hospitals Leuven, Leuven, Belgium.; 3 Department of Gynecological and Obstetrics, University Hospitals Leuven, Leuven, Belgium.; 4 Department of Medical Oncology, Institut Curie, Saint Cloud, France.; 5 Department of Medical Oncology, Centre Henri Becquerel, Rouen, France.; 6 Anatomical Pathology Unit, Department of Biopathology, Centre Henri Becquerel, Rouen, France.; 7 Department of Internal Medicine and Medical Specialties (DiMI), School of Medicine, University of Genova, Genova, Italy.; 8 Department of Medical Oncology, U.O. Clinica di Oncologia Medica, IRCCS Ospedale Policlinico San Martino, Genova, Italy.; 9 Anatomical Pathology Unit, Department of Surgical Sciences and Integrated Diagnostics, University of Genova, Genoa, Italy.; 10 Department of Internal Medicine and Specialistic Medicine, U.O. Medicina Interna a Indirizzo Oncologico, IRCCS Ospedale Policlinico San Martino, Genova, Italy.; 11 Translational Cancer Research Unit, Center for Oncological Research, Faculty of Medicine and Health Sciences, University of Antwerp, GZA hospitals, Antwerp, Belgium.; 12 Department of General Medical Oncology, University Hospitals Leuven, Leuven, Belgium.; 13 Department of Surgical Oncology, University Hospitals Leuven, Leuven, Belgium.; 14 Department of Gynecological Oncology, University Hospitals Leuven, Leuven, Belgium.; 15 Department of Pathology, Université Paris Sciences Lettres, Institut Curie, Paris, France.; 16 Department of Pathology, Institut Jules Bordet, Brussels, Belgium.; 17 Clinique du sein, Centre Hospitalier du Luxembourg, Luxembourg.; 18 Institut Paoli-Calmettes, Marseille, France.; 19 Unit of Medical Statistics, Biometry and Epidemiology, Department of Biomedical and Clinical Sciences (DIBIC) “L. Sacco” & DSRC, LITA Vialba campus, University of Milan, Milan, Italy.; 20 Laboratory for Translational Cell and Tissue Research, Department of Pathology and Imaging, KU Leuven, Belgium.

## Abstract

Inflammatory breast cancer (IBC) is a rare (1%–5%), aggressive form of breast cancer, accounting for approximately 10% of breast cancer mortality. In the localized setting, standard of care is neoadjuvant chemotherapy (NACT) ± anti-HER2 therapy, followed by surgery. Here we investigated associations between clinicopathologic variables, stromal tumor-infiltrating lymphocytes (sTIL), and pathologic complete response (pCR), and the prognostic value of pCR. We included 494 localized patients with IBC treated with NACT from October 1996 to October 2021 in eight European hospitals. Standard clinicopathologic variables were collected and central pathologic review was performed, including sTIL. Associations were assessed using Firth logistic regression models. Cox regressions were used to evaluate the role of pCR and residual cancer burden (RCB) on disease-free survival (DFS), distant recurrence-free survival (DRFS), and overall survival (OS). Distribution according to receptor status was as follows: 26.4% estrogen receptor negative (ER^−^)/HER2^−^; 22.0% ER^−^/HER2^+^; 37.4% ER^+^/HER2^−^, and 14.1% ER^+^/HER2^+^. Overall pCR rate was 26.3%, being highest in the HER2^+^ groups (45.9% for ER^−^/HER2^+^ and 42.9% for ER^+^/HER2^+^). sTILs were low (median: 5.3%), being highest in the ER^−^/HER2^−^ group (median: 10%). High tumor grade, ER negativity, HER2 positivity, higher sTILs, and taxane-based NACT were significantly associated with pCR. pCR was associated with improved DFS, DRFS, and OS in multivariable analyses. RCB score in patients not achieving pCR was independently associated with survival. In conclusion, sTILs were low in IBC, but were predictive of pCR. Both pCR and RCB have an independent prognostic role in IBC treated with NACT.

**Significance:**

IBC is a rare, but very aggressive type of breast cancer. The prognostic role of pCR after systemic therapy and the predictive value of sTILs for pCR are well established in the general breast cancer population; however, only limited information is available in IBC. We assembled the largest retrospective IBC series so far and demonstrated that sTIL is predictive of pCR. We emphasize that reaching pCR remains of utmost importance in IBC.

## Introduction

Inflammatory breast cancer (IBC) is a rare type of breast cancer, with a prevalence of 1%–5% (1) in Western countries and a relatively higher prevalence in the African American (1, 2) and North African population (7%–11.1%; refs. 3, 4). IBC accounts for 10% of all breast cancer mortality, with a median overall survival of 4.2 years (5). Up to 56% to 84% of the patients with IBC have axillary lymph node involvement (6–8) and up to 25% of the patients have metastatic disease at diagnosis (9), underpinning the aggressive behavior of IBC.

IBC is a clinical diagnosis, characterized by criteria described by Dawood and colleagues (10). However, the pathologic confirmation of invasive carcinoma remains essential (10). The term “inflammatory” in IBC might be misleading since there is no, or little inflammation involved on microscopic examination. It instead refers to the inflammatory clinical presentation of edema, redness, and enlargement of the breast, which is thought to be caused by obstruction of dermal lymphatic vessels of the breast by tumor emboli. Tumor emboli in skin biopsies are observed in up to 75% of cases but do not represent a mandatory diagnostic criterion for IBC (11, 12).

IBC is presented with a higher percentage of the more aggressive surrogate molecular subtypes in comparison with the non-IBC population. Within IBC, 24% are reported to be triple negative (11, 13) and 32% to 45% are HER2^+^ (11, 13–15) in contrast to 10% to 15% of triple-negative and 13% to 15% of HER2^+^ cases for the non-IBC population (16). The median age of diagnosis is generally lower for IBC in comparison with locally advanced non-IBC (57.3 years for IBC vs. 64.3 years for locally advanced non-IBC; refs. 13, 14).

Because of the aggressive behavior, the international expert panel on IBC recommends trimodal therapy consisting of anthracycline- and taxane-based neoadjuvant chemotherapy (NACT), followed by mastectomy and radiotherapy, in the localized setting (10). Anti-HER2 therapy is given in case of *HER2*^+^ tumors (10). Postoperatively, adjuvant systemic therapy may be delivered according to the stage at diagnosis and degree of pathologic response, and endocrine therapy is administered when indicated. However, despite this multimodal treatment, and improvement of systemic treatments over time, survival rates are still generally lower as compared with stage-matched non-IBC tumors (8, 11, 17–19). Novel treatments are also being implemented for IBC; however, this has been extrapolated from studies investigating non-IBC, such as addition of pembrolizumab in neoadjuvant and metastatic setting for triple-negative IBC and the use of adjuvant T-DM1 for HER2^+^ IBC (20).

Response to NACT in breast cancer can be measured on the resection specimen by pathologic complete response (pCR), defined by the absence residual invasive carcinoma in the breast and lymph nodes (ypT0 or ypTis and ypN0 according to TNM classification (21). This is the most acknowledged one, following the international recommendations (22) and is independently associated with prognosis in the general breast cancer population (23, 24). In patients with IBC, studies indicate that pCR rates vary between 9% and 40% and depend on the (surrogate) molecular subtype (18, 19, 25–27). While pCR has been associated with an improved outcome for patients with IBC, survival rates are inferior to stage-matched patients with non-IBC (11, 18, 19, 26). Large retrospective studies have now established the value of stromal tumor infiltrating lymphocytes (sTIL) to predict pCR to NACT in all surrogate molecular subtypes of the general breast cancer population (28). Higher sTIL levels were, however, only associated with better prognosis in patients with triple-negative and HER2^+^ breast cancer. For IBC, this has only been sporadically investigated thus far, and often in relatively small cohorts (19, 26, 27).

A standardized method for evaluation of response to NACT was developed under the form of the residual cancer burden (RCB) score (29), which entails a continuous score as well as a categorical variable. An online tool calculates the score and class based on size of tumor bed, residual tumor size and cellularity, percentage of *in situ* carcinoma, number of positive lymph nodes and maximum diameter of the lymph node metastasis (30). Notably, the RCB is based on different measurements of residual disease as compared with standard staging by TNM. Both RCB-class and continuous RCB-scores have been associated with outcome with higher RCB-class and RCB-score resulting in worse outcomes for all patients as well as within the different molecular subtypes (24, 29). This has not been reported so far specifically in patients with IBC.

In this study, we assembled to our knowledge the largest multicentric retrospective cohort of patients with IBC with extensive clinical data collection, coupled with central pathologic review. We aimed at evaluating which variables, including sTILs, are associated with response to NACT, quantified by pCR and RCB, and determining the prognostic value of pCR in patients with IBC.

## Materials and Methods

### Patient Population

This study included a retrospective, European multicentric cohort of female patients diagnosed with IBC and treated between October 1996 and October 2021 at eight different European hospitals (GZA hospitals Antwerp, Institut Jules Bordet Brussels, University Hospitals Leuven, Centre Henri Becquerel Rouen, Institut Curie Paris and Saint Cloud, Institut Paoli-Calmettes Marseille, IRCCS Policlinico San Martino Genova, Centre Hospitalier du Luxembourg). Patients were selected on the basis of the reported cT4d T-stage according to the TNM classification (21, 31). The clinical files were then refined for the clinical criteria defined by Dawood and colleagues, to discriminate between “real” IBC and secondary IBC, that is, IBC-like presentation due to progression of a neglected breast cancer and/or ulceration (10). Only patients who received NACT with or without anti-HER2 therapy, followed by surgery of the primary tumor, were eligible for this study. For the purpose of our research question, patients who only received neoadjuvant endocrine therapy were not included.

A data collection template was set-up centrally to collect clinicopathologic, treatment, and survival data, and was distributed to clinicians of the participating centers. Collected data were inspected for inconsistencies and potential input errors, which were rectified, before being consolidated into a central database. Lymph node status was recorded as the clinically assessed (cN) before neoadjuvant chemotherapy. BMI was stratified into underweight (<18.5 kg/m^2^), lean (18.5–25 kg/m^2^), overweight (25–30 kg/m^2^) and obese (≥30 kg/m^2^) according to the WHO expert committee (32). Menopausal status was provided by the participating centers in accordance with the medical records. For the purpose of this study, patients were categorized in four subgroups by the status of estrogen receptor (ER) and HER2 (ER^−^/HER2^−^, ER^−^/HER2^+^, ER^+^/HER2^−^, and ER^+^/HER2^+^) determined by locally performed IHC ± *in situ* hybridization for the latter. ER was considered positive if Allred score was >1% and if H-score >0. In case no continuous score was provided, the historical status (positive vs. negative) was used if available. Analyses were stratified according to the subgroups.

### Central Pathology Review

The archived diagnostic formalin-fixed paraffin-embedded (FFPE) biopsies were retrieved for central pathology review, including a total number of 302 breast tumor biopsies, 16 skin biopsies, and 46 biopsies of breast tumor with overlying skin (Supplementary Table S1). Most of them, 315 samples, were core needle biopsies, with availability of 50 larger incisional biopsies. The hematoxylin and eosin (H&E)-stained slides were reviewed and consensus scoring by G. Floris, P. Vermeulen, and M. De Schepper, as performed in a systematical manner according to the The Cancer Genome Atlas pathology data sheet” (TCGA) adapted to IBC by including scoring of the presence of tumor emboli (33, 34).

sTILs were scored according to the guidelines of the international TILs working group (35), and evaluated in three representative fields. The mean of these three fields was used as sTIL level at a sample level. In case multiple samples were available, the average sTIL of all fields across all available samples was used at a patient level. In subsequent analyses, sTILs were considered both as a continuous variable, and as a categorical variable of three categories – low (sTIL score ≤ 10%), intermediate (sTIL score > 10% and < 60%), and high (sTIL score ≥ 60%; ref. 28). sTILs were scored both on tumor samples of the breast as well as breast skin samples. sTIL scoring on the latter was performed only in the invasive carcinoma according to the guidelines, hereby excluding the stroma surrounding the tumor emboli (ref. 36; Supplementary Fig. S1).

Tumor emboli were examined surrounding the invasive carcinoma and at distance in the skin in case skin biopsies were present at time of diagnosis. Density of emboli was determined by counting the maximum number of identifiable emboli under the microscope with magnification of 200× and field diameter of 1.25 mm.

Response to neoadjuvant systemic therapy was measured using pCR (defined as ypT0/is ypN0) and residual cancer burden (RCB) class (0 = pCR; I; II and III) as well as the continuous score (29). As RCB scores were systematically assessed only for patients diagnosed and treated at the University Hospitals Leuven, all analyses involving RCB were done strictly in this sub-cohort (Supplementary Table S2). Because of a relatively limited number of patients, these analyses were not done in the breast cancer subtypes.

Other parameters that were analyzed were retraction clefts and stromal reaction. Retraction clefts were evaluated on routine H&E section as optical empty spaces between tumor cells and stroma. This was scored binary with a cutoff of 20% according to Acs and colleagues (37). Stromal reaction pattern was scored in accordance with the TCGA scoring sheet (34).

### Statistical Analysis

For cases with multiple biopsies sites per patient (tumor biopsy and skin biopsy), the concordance of sTIL score and tumor emboli between the two was first inspected using the Bland–Altman and Lin's Concordance Correlation Coefficient (CCC) methods.

Linear regression models were used to investigate the association between sTILs, as the continuous dependent variable, and each of the standard clinicopathologic features. Associations between pCR, as a binary dependent variable, and each of the clinicopathologic features, as well as sTILs, were assessed using Firth logistic regression models. With RCB class as dependent variable, association analyses were performed using multinomial logistic regression models with RCB-0 as baseline. Furthermore, the impact of clinicopathologic features on the extent of residual disease in patients who did not achieve pCR, that is, RCB nonzero score, was inspected by analyzing the association of continuous RCB score with these variables using linear regression models performed on the corresponding subset of patients.

The three endpoints considered in this study are disease-free survival (DFS), distant recurrence-free survival (DRFS), and overall survival (OS). DFS was defined as the duration from diagnosis to the first event of either locoregional, contralateral, or distant recurrence, or death from any cause; DRFS as the duration from diagnosis to the first event of distant recurrence; and OS as the duration from primary diagnosis to death from any cause. The reverse Kaplan–Meier (KM) method was used to estimate the median follow-up. KM curves were constructed to inspect the probabilities of DFS and OS in patients with and without pCR, or in patients of different RCB classes. Cox regression models were used to evaluate the effect of either pCR, RCB class, or RCB score on DFS and OS. DRFS was analyzed considering death without distant recurrence as the competing event. Crude cumulative incidence curves accounting for the competing risk were used to estimate the event rates of this endpoint according to pCR status and RCB class. Regression analyses of DRFS were performed using Fine–Gray regression models.

For each of the abovementioned regression analyses, two models were used. Model 1 assessed the association between the dependent variable and a single explanatory variable. Model 2 considered all clinicopathological variables of interest, and treatment where applicable, as independent variables. Both models accounted for the heterogeneity between centers by adjustment or stratification. In the text, Model 1 and Model 2 will be further referred to as univariable and multivariable models for simplicity.

Quantile regression was used to assess the potential non-linear associations between continuous dependent variables and categorical independent variables. In cases where the independent variable of interest was a continuous variable, a restricted cubic spline was used in the regression models to allow for nonlinear effects. A likelihood ratio (LR) test was then used for the comparative performance evaluation of the linear models and the models with nonlinear effects. Potential nonlinear associations suggested by these exploratory analyses are reported where appropriate.

Statistical analyses were executed using R version 4.1.1. A two-sided *P* value < 0.05 was used as the standard evidence criterion of statistical significance for all statistical tests. All *P* values reported were not corrected for multiple testing.

### Ethics Statement

#### Ethics Approval

This study was approved by the central ethics committee of University hospitals of Leuven (S62499) on December 20, 2019. No informed consent form (ICF) was requested as a waiver was granted given that many patients already passed away or progressed. The study was conducted in accordance with the Declaration of Helsinki.

### Data Availability Statement

The data generated in this study are not publicly available due to restrictions of the protocol approved by the ethics committees of the involved hospitals. Access to the data can be requested via the corresponding author.

## Results

### Presentation of the Study Cohort

A total of 494 patients with nonmetastatic IBC were included. The breast cancer subtype based on the ER status and HER2 status was available for 454 patients: 26.4% (120/454) were ER^−^/HER2^−^, 22.0% (100/454) ER^−^/HER2^+^, 37.4% (170/454) ER^+^/HER2^−^, and 14.1% (64/454) ER^+^/HER2^+^ (Table 1). Invasive breast cancer of no special type (IBC-NST, formerly called ductal breast cancer) was the dominant histologic type in the full study population (91.3%, 400/494) and in the respective subgroups (Table 1). In the entire cohort, most tumors were grade 3 (64.6%, 285/494), with relatively lower number of grade 3 tumors in the ER^+^/HER2^−^ subgroup (46.5%, 73/157) as compared with the other subgroups (Table 1). Most patients had locoregional lymph node involvement at time of diagnosis (81.6%, 389/477 of all patients), without significant differences between the subgroups (Table 1). Of note, a center effect was observed with relatively lower number of patients having a clinical positive lymph node status at diagnosis in Institut Curie Paris (65%, 33/51) in comparison to GZA hospitals Antwerp (82%, 63/77). Most of the patients received taxane-based NACT (79.7%, 384/482), followed by mastectomy (96.8%, 367/379), and radiotherapy (96.7%, 412/426) with no differences in the treatment scheme between the different subgroups. Within the HER2^+^ groups, 35.9% (61/170) did not receive neoadjuvant anti-HER2 therapy (40%, 40/100 for ER^−^/HER2^+^; and 32.8%, 21/64 for ER^+^/HER2^+^ subgroups; Table 1). The majority of these patients (95.1%, 58/61) were diagnosed before 2010, when the neoadjuvant anti-HER2 treatment has become standard of care for these patients.

**TABLE 1:** Baseline clinical and pathologic characteristics of patients in the entire cohort and in each of the breast cancer subgroups

		All *N* (%)	ER^−^/HER2^−^*N* (%)	ER^−^/HER2^+^*N* (%)	ER^+^/HER2^−^*N* (%)	ER^+^/HER2^+^*N* (%)	*P*
Total patients		494 (100)	120 (26.4)	100 (22.0)	170 (37.4)	64 (14.1)	
Age	≤50	194 (39.3)	49 (40.8)	32 (32.0)	65 (38.2)	30 (46.9)	0.272
	>50	300 (60.7)	71 (59.2)	68 (68.0)	105 (61.8)	34 (53.1)	
Menopausal status	Pre/Perimenopausal	192 (41.8)	49 (43.4)	32 (34.0)	67 (41.6)	27 (44.3)	0.512
	Postmenopausal	267 (58.2)	64 (56.6)	62 (66.0)	94 (58.4)	34 (55.7)	
	Unknown	35	7	6	9	3	
BMI category	Underweight (<18.5 kg/m^2^)	3 (0.6)	0 (0.0)	2 (2.1)	1 (0.6)	0 (0.0)	0.372
	Lean (18.5–25 kg/m^2^)	165 (35.4)	37 (33.9)	38 (40.0)	57 (34.8)	19 (30.2)	
	Overweight (25–30 kg/m^2^)	159 (34.1)	39 (35.8)	35 (36.8)	52 (31.7)	20 (31.7)	
	Obese (≥30 kg/m^2^)	139 (29.8)	33 (30.3)	20 (21.1)	54 (32.9)	24 (38.1)	
	Unknown	28	11	5	6	1	
Focality	Unifocal	230 (79.6)	50 (82.0)	48 (78.7)	78 (79.6)	34 (75.6)	0.893
	Multifocal	59 (20.4)	11 (18.0)	13 (21.3)	20 (20.4)	11 (24.4)	
	Unknown	205	59	39	72	19	
Histology	ILC	30 (6.9)	6 (5.9)	2 (2.3)	14 (9.5)	5 (7.9)	0.301
	NST	400 (91.3)	95 (93.1)	84 (96.6)	129 (87.2)	57 (90.5)	
	Other	8 (1.8)	1 (1.0)	1 (1.1)	5 (3.4)	1 (1.6)	
	Unknown	56	18	13	22	1	
Grade	G1	15 (3.4)	3 (2.8)	0 (0.0)	9 (5.7)	2 (3.3)	<0.001
	G2	141 (32.0)	13 (12.3)	23 (24.7)	75 (47.8)	22 (36.1)	
	G3	285 (64.6)	90 (84.9)	70 (75.3)	73 (46.5)	37 (60.7)	
	Unknown	53	14	7	13	3	
ER status	Negative	229 (48.5)	120 (100.0)	100 (100.0)	0 (0.0)	0 (0.0)	*NA*
	Positive	243 (51.5)	0 (0.0)	0 (0.0)	170 (100.0)	64 (100.0)	
	Unknown	22	0	0	0	0	
HER2 status	Negative	297 (63.6)	120 (100.0)	0 (0.0)	170 (100.0)	0 (0.0)	*NA*
	Positive	170 (36.4)	0 (0.0)	100 (100.0)	0 (0.0)	64 (100.0)	
	Unknown	27	0	0	0	0	
PR status	Negative	295 (64.6)	114 (97.4)	93 (95.9)	51 (31.9)	22 (35.5)	<0.001
	Positive	162 (35.5)	3 (2.6)	4 (4.1)	109 (68.1)	40 (64.5)	
	Unknown	37	3	3	10	2	
Lymph node positivity	No	88 (18.5)	22 (18.6)	13 (13.5)	35 (20.7)	11 (17.2)	0.544
	Yes	389 (81.6)	96 (81.4)	83 (86.5)	134 (79.3)	53 (82.8)	
	Unknown	17	2	4	1	0	
Neoadjuvant anti-HER2	No	368 (76.7)	115 (99.1)	40 (40.0)	161 (98.2)	21 (32.8)	<0.001
	Yes	112 (23.3)	1 (0.9)	60 (60.0)	3 (1.8)	43 (67.2)	
	Unknown	14	4	0	6	0	
Neoadjuvant chemotherapy scheme	Taxane	384 (79.7)	89 (76.7)	82 (82.0)	139 (83.7)	53 (82.8)	0.527
	No Taxane	98 (20.3)	27 (23.3)	18 (18.0)	27 (16.3)	11 (17.2)	
	Unknown	12	4	0	4	0	
Surgery	Mastectomy	367 (96.8)	81 (94.2)	79 (100.0)	122 (96.1)	52 (100.0)	0.067
	Tumorectomy	12 (3.2)	5 (5.8)	0 (0.0)	5 (3.9)	0 (0.0)	
	Unknown	115	34	21	43	12	
Radiotherapy	No	14 (3.3)	4 (4.2)	2 (2.3)	7 (4.8)	1 (1.6)	0.6752
	Yes	412 (96.7)	91 (95.8)	85 (97.7)	139 (95.2)	62 (98.4)	
	Unknown	68	25	13	24	1	
pCR	No pCR	355 (73.7)	81 (70.4)	53 (54.1)	152 (89.9)	36 (57.1)	<0.001
	pCR	127 (26.4)	34 (29.6)	45 (45.9)	17 (10.1)	27 (42.9)	
	Unknown	12	5	2	1	1	
RCB class^#^	0	40 (28.2)	8 (57.1)	18 (42.8)	2 (4.2)	11 (4.8)	<0.001
	I	12 (8.4)	1 (7.1)	5 (11.9)	2 (4.2)	4 (17.4)	
	II	45 (31.7)	2 (14.3)	14 (33.3)	19 (39.6)	6 (26.1)	
	III	45 (31.7)	3 (21.4)	5 (11.9)	25 (52.1)	2 (8.7)	
	Unknown	21	14	0	7	0	

^#^Statistics are described for patients diagnosed and treated at the University Hospitals Leuven.

### Clinicopathologic Characteristics Based on Central Pathology Review

Central pathology review was performed on 366 unique samples accounting for 68.4% (338/494) of the cases with clinical information (Supplementary Table S1). Reasons for absent central review were missing samples from the historical archives or insufficient sample quality (Supplementary Fig. S2). Insufficient sample quality was characterized by faded H&E staining precluding morphologic interpretation. Clinicopathologic variables did not differ between the group of patients with evaluable biopsy and the group without (Supplementary Table S1).

**FIGURE 1:**
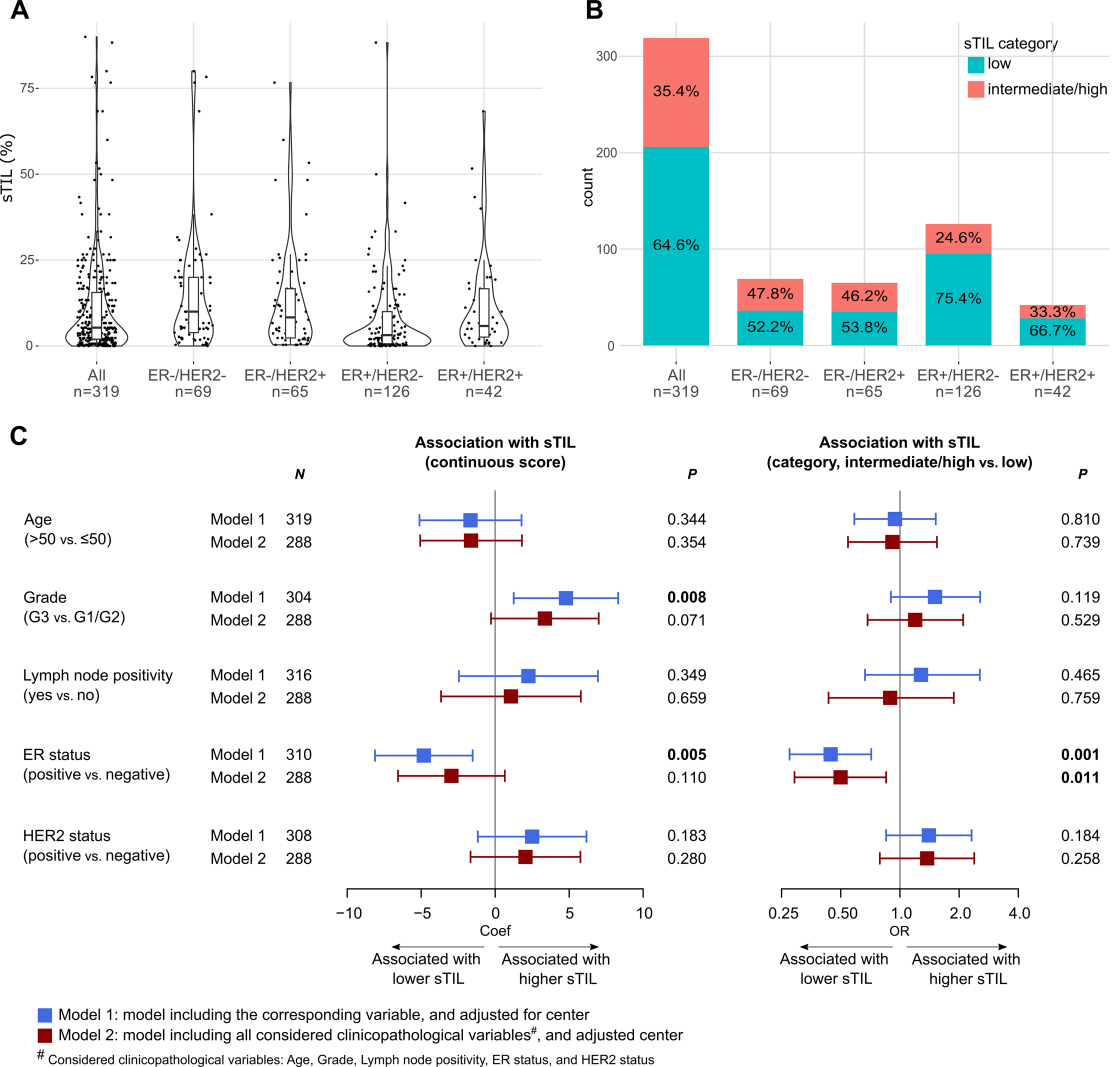
sTIL scoring and its association with clinicopathological features in all patients. **A,** Distribution of sTIL (continuous) in the entire cohort and in subtypes. **B,** Distribution of sTIL (category) in the entire cohort and in subtypes. **C,** Forest plots showing the association of sTIL (continuous) and sTIL (categorical) with standard clinicopathologic variables evaluated by regression analyses in all patients.

sTILs were scored for 94.4% (319/338) patients, among whom matched tumor and skin biopsies were available for 8 patients. Concordance analysis of these samples indicated a strong concordance in sTIL scoring between both biopsy sites for all patients (Supplementary Fig. S3). The average sTIL score of all samples from the same patient was therefore used at the patient's level in the subsequent analyses. Median sTILs was 5.3% [IQR (2.0%–15.6%)] and most patients (64.6%, 206/319) had low sTILs, defined as ≤10% (Fig. 1A and B). ER^−^ tumors tended to have a higher sTIL level compared with ER^+^ tumors [median of 10.0%, IQR (3.9–20.0) in ER^−^/HER2^−^; and 8.3%, IQR (2.3–16.7) in ER^−^/HER2^+^; versus 3.2%, IQR (0.7–10.0) in ER^+^/HER2^−^; and 5.8%, IQR (2.6–16.7) in ER^+^/HER2^+^; Fig. 1A and B]. Given few cases with sTILs belonging to the “high” (≥60%) category (8 cases in the entire cohort, 4 in ER^−^/HER2^−^, and 1 in each of the remaining subgroups), we considered two categories of sTIL, “low” and “intermediate/high”, in subsequent analyses.

Tumor emboli from peritumoral samples were identified for 19.8% (67/338) patients (Supplementary Fig. S4A). Among patients for whom skin biopsies were available, emboli were present in 49.2% (30/61) patients. In contrast to sTILs, there was a discordance between the levels of tumor emboli detected in tumor and skin biopsies, which however presented no clear orientation of favoring a biopsy site (Supplementary Fig. S4B and S4C). The tumor emboli were therefore separately described for tumor and skin biopsies (Supplementary Fig. S4D).

### Association of sTILs with Clinicopathologic Features

Univariable regression analyses showed that higher sTILs was associated with high-grade and ER^−^ tumors. This corresponds to the observed differences in sTIL level between subtypes. Only a trend was retained in the multivariable model (Fig. 1C). Quantile regressions revealed potentially nonlinear associations of several clinicopathologic variables with sTILs along its range, especially for grade, ER status, and HER2 status (Supplementary Fig. S5). However, no opposite associations to those detected by the linear analysis were observed.

Descriptive data suggested that sTILs tended to be lower in ILC tumors compared with subgroup-matched NST tumors although with a lack of statistical evidence due to very few cases of ILC (Supplementary Table S3). Missing data and unfeasibility to confirm histologic diagnosis with central pathology also hindered the evaluation with regard to this feature, therefore histologic subtype was excluded in our current multivariable analyses. Subgroup analyses did not provide evidence of an association between sTILs and clinicopathologic variables of interest in the two ER^+^ subgroups (Supplementary Fig. S6). In the ER^−^ subgroups, opposite patterns according to HER2 status in terms of relationship with sTILs were observed for lymph node (LN) status. sTILs tended to be higher in LN-positive compared with LN-negative ER^−^/HER2^−^ patients, while it was significantly higher in LN-negative compared with LN-positive ER^−^/HER2^+^ patients (Supplementary Fig. S6). Similarly, opposite trends were seen for the association of the continuous sTIL score and age, yet this was not reflected in the analyses considering sTIL as categorical variable. Nonlinear relationship was not assessed due to a limited number of cases in each of these subgroups.

### Response to NACT and its Association with Clinicopathologic Features

**FIGURE 2:**
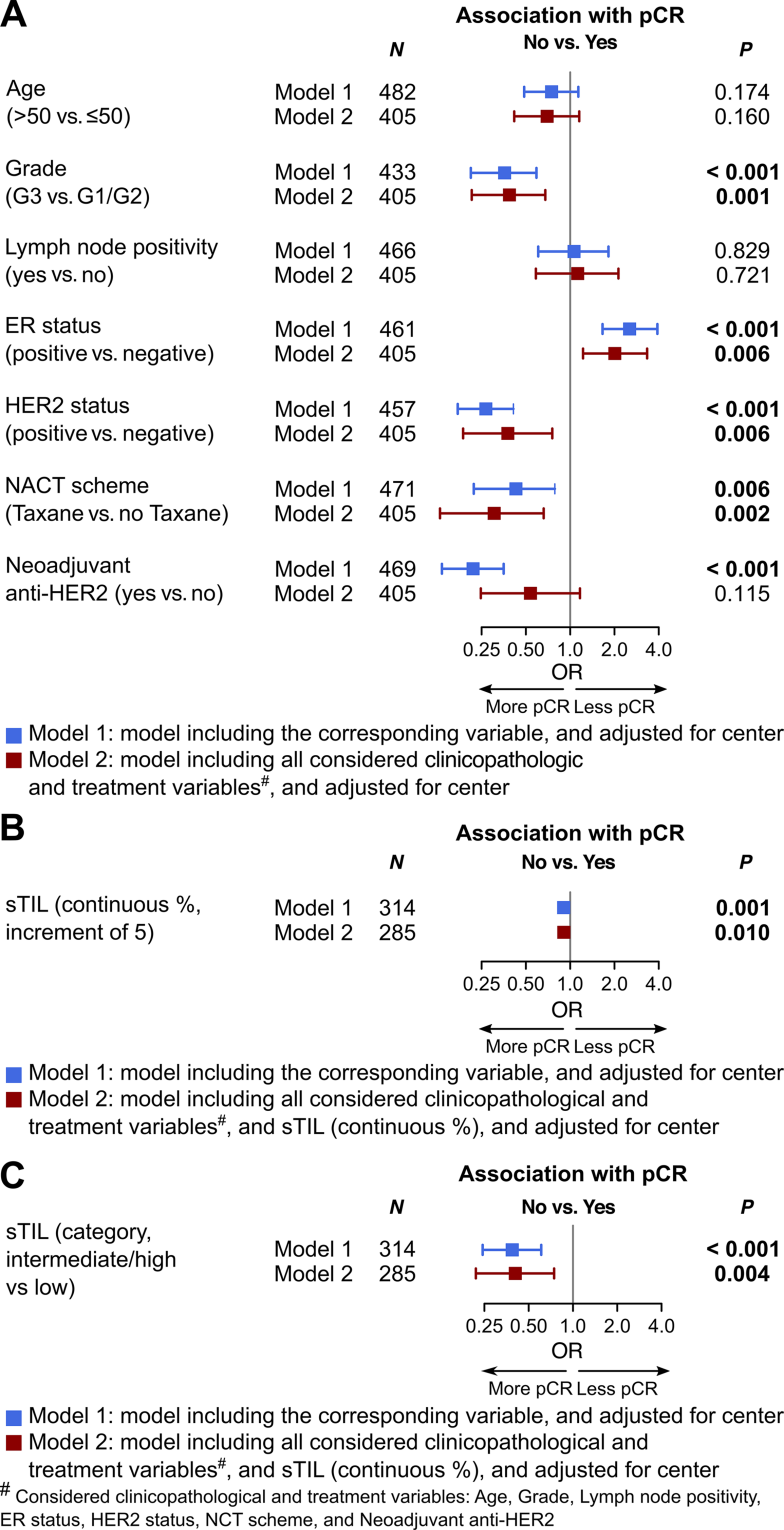
Association of pCR with clinicopathologic features and treatment in all patients. **A–C,** Forest plots showing the association of pCR with standard clinicopathologic and treatment variables (A), with sTILs (continuous %; B), and with sTILs (categorical; C) evaluated by regression analyses in all patients.

pCR was reached in 26.3% (127/482) patients, with the highest rates observed in the HER2^+^ groups (45.9%, 45/98 for ER^−^/HER2^+^; and 42.9%, 27/63 for ER^+^/HER2^+^), followed by 29.6% (34/115) in the ER^−^/HER2^−^ group, and 10.1% (17/169) in the ER^+^/HER2^−^ group (Table 1). A higher grade, ER^−^ status, HER2^+^ status, taxane-based NACT, and anti-HER2 therapy were associated with pCR considering all patients (Fig. 2A). In subgroup analyses, these associations were recapitulated, but only for certain subgroups. Specifically, grade and NACT scheme were associated with pCR in the HER2^+^ subgroups (Supplementary Fig. S7A). Within the HER2^+^ subgroups, pCR rates were numerically higher in patients receiving neoadjuvant anti-HER2 therapy versus no neoadjuvant anti-HER2 therapy [52.4% (54/103) vs. 30.2% (19/63)], and similarly when stratifying according to ER status: 57.9% (33/57) versus 29.3% (12/41) in the ER^−^/HER2^+^ subgroup and 48.8% (20/41) versus 31.8% (7/22) in the ER^+^/HER2^+^ subgroup. All patients receiving anti-HER2 therapy also received taxane containing NACT. For patients with HER2^+^ disease not receiving anti-HER2 therapy, benefit of taxanes was clearly seen with pCR rate of 50% (16/32) in the taxane group and 20.7% (6/29) in the group without taxanes. Patients of age 50 years and below in the ER^−^/HER2^+^ and ER^+^/HER2^−^ subgroups were more likely to achieve pCR, while in the ER^−^/HER2^−^ subgroup, a trend toward the opposite direction was seen (Supplementary Fig. S7A). Higher sTILs were independently associated with pCR in the whole cohort (Fig. 2B and C). However, when subtype was taken into consideration, increase in sTILs (when considered as categorical variable) resulted in an increased odds of achieving pCR in the two ER^−^ subgroups, albeit with weak statistical evidence (Supplementary Fig. S7B and S7C). We also explored several other pathologic features of IBC tumors such as tumor emboli, for their associations with pCR but did not observe any notable patterns (Supplementary Fig. S8).

**FIGURE 3:**
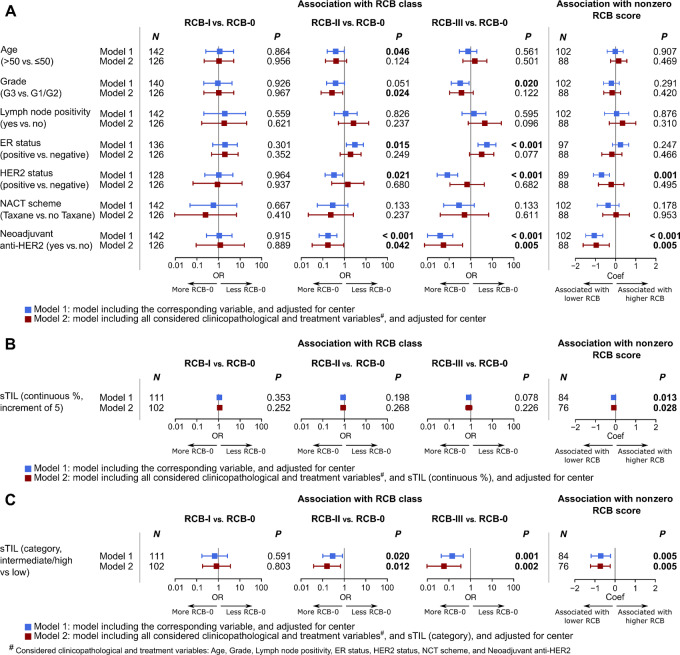
Association of RCB with clinicopathologic features and treatment in all patients. **A–C,** Forest plots showing the association of RCB, either as RCB class or continuous RCB score, with standard clinicopathologic and treatment variables (A), with sTILs (continuous %; B), and with sTILs (categorical; C) evaluated by regression analyses in all patients. Analyses were performed on the subset of patients diagnosed and treated at UZ Leuven, Belgium.

RCB scores were available for 142 patients (Leuven cohort, Supplementary Table S2). This subcohort did not differ significantly in clinicopathologic variables from the entire cohort, except for taxane-based NACT, which was higher in the Leuven cohort than in the total cohort [89.6% (146/163) vs. 79.7% (384/482)] and tumor focality, with relative higher unifocal tumors in the Leuven subcohort [87.7% (143/163) vs. 79.6% (230/289) respectively]. In this subcohort, patients in the ER^−^/HER2^−^ subgroup achieved the highest rate of RCB-0 (pCR, *P* < 0.001; Table 1). On the contrary, the ER^+^/HER2^−^ subgroup had both the lowest rate of RCB-0 and highest rate of RCB-III. No differences in clinicopathologic characteristics between RCB-I and RCB-0 (pCR) patients were observed (Fig. 3A, first column). The differences in baseline characteristics and treatment of patients with extensive residual disease, that is, RCB-II and RCB-III, and patients achieving RCB-0 (pCR) were similar to those found by the analyses regarding no-PCR versus pCR respectively (Fig. 3A, second and third columns). Considering only cases with residual disease, that is, nonzero RCB score, only the use of neoadjuvant anti-HER2 treatment was independently associated with a lower RCB extent of the residual disease (Fig. 3A, fourth column). Similar to pCR, higher sTILs seemed to be predictive of a lower RCB; however, the differences were more evident when comparing intermediate/high sTIL versus low sTIL (Fig. 3B and C). It was suggested by exploratory analyses that the association between RCB score and sTILs in patients with residual disease, that is, nonzero RCB score, could potentially be nonlinear (Supplementary Fig. S9), which needs further investigation in larger data series. In our current data, their correlation however remained positive throughout the range of sTILs as reflected by positive coefficient estimates.

### Association of Response to NACT with Prognosis

**FIGURE 4:**
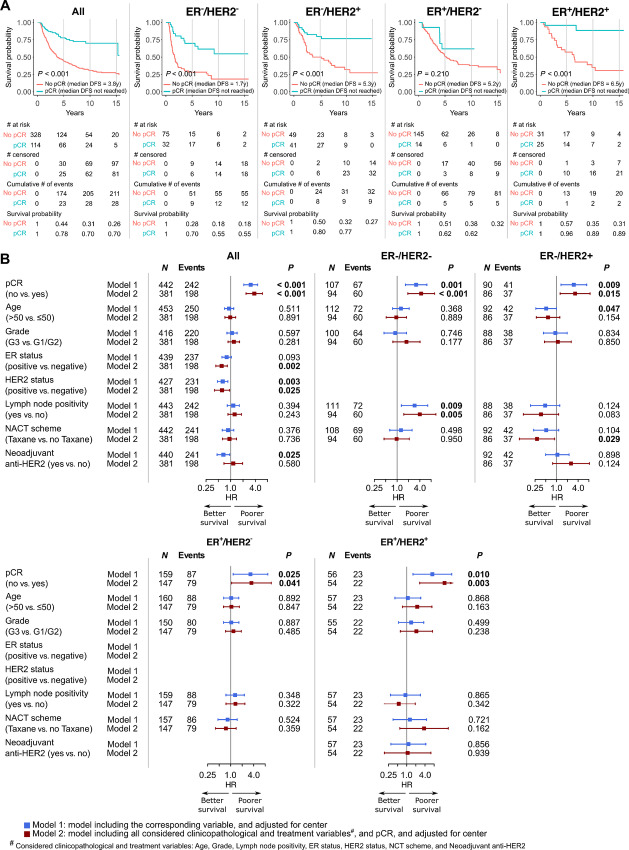
Association of pCR with DFS. **A,** Kaplan–Meier curves of DFS according to pCR. **B,** Forest plots showing the association of pCR and standard clinicopathologic and treatment variables with DFS quantified by Cox regression.

**FIGURE 5:**
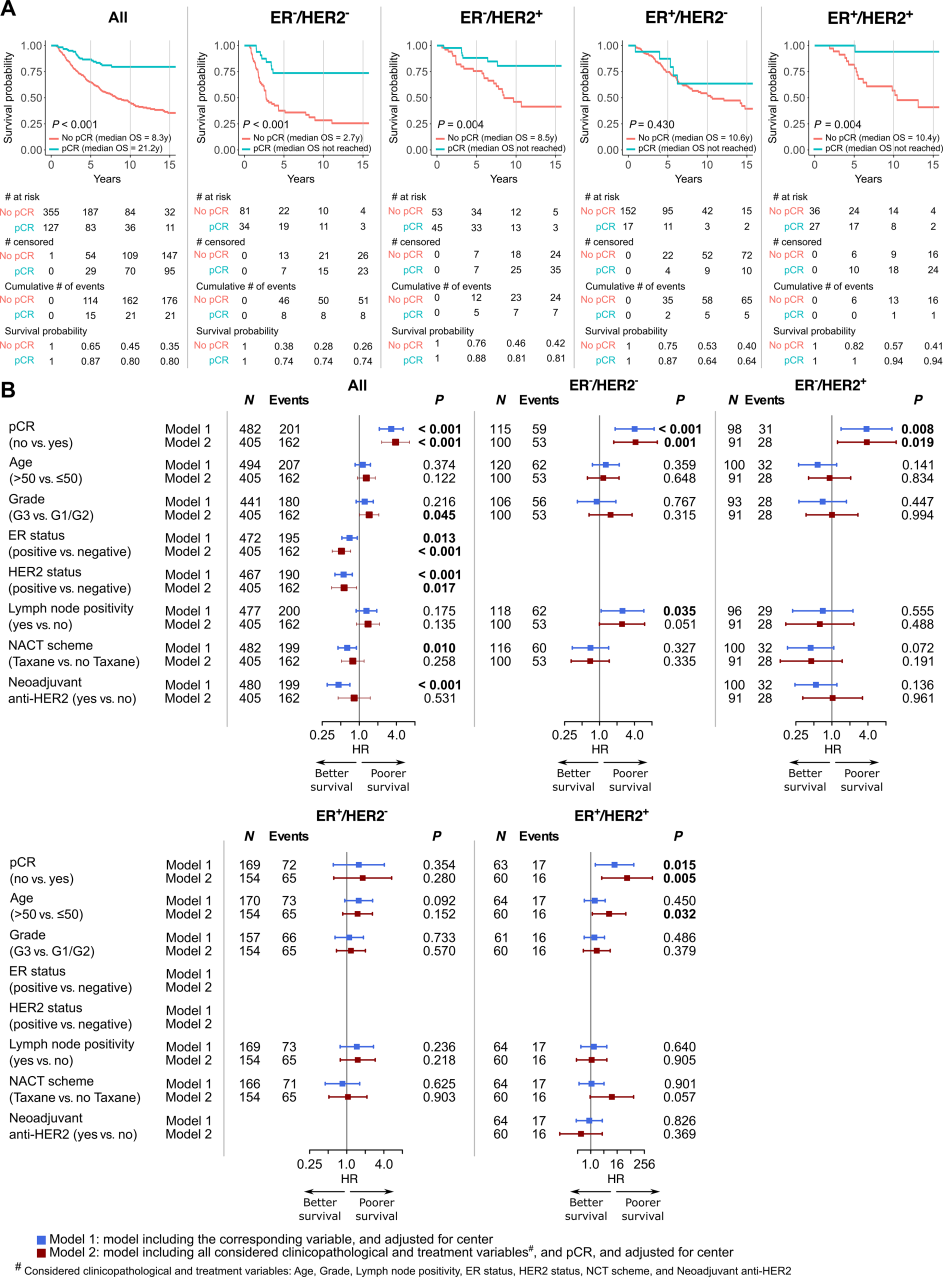
Association of pCR with OS. **A,** Kaplan–Meier curves of OS according to pCR. **B,** Forest plots showing the association of pCR and standard clinicopathologic and treatment variables with OS quantified by Cox regression.

The median follow-up duration, median DFS, and median OS of all patients were 9.4, 5.8, and 11.6 years, respectively. Both univariable and multivariable analyses revealed that achieving pCR was generally associated with better DFS, DRFS, and OS in all patients, without and with consideration of subtype (Figs. 4 and 5; Supplementary Fig. S10). The survival benefit of achieving pCR was less extensive for the ER^+^/HER2^−^ subtype compared with other subtypes. RCB classes were also shown to be prognostic for all endpoints (Supplementary Fig. S11 and S12). Of note, improved OS in patients achieving RCB-0 compared with patients with extensive residual disease, that is, RCB-III, was apparent throughout the maximum follow-up duration, while patients with less extensive or minimal residual disease, that is, RCB-II and RCB-I, started to show a noticeably worse OS at approximately 5 years of follow-up (Supplementary Fig. S11D).

The independent prognostic role of RCB score in patients not achieving pCR, that is, nonzero RCB score, was also shown with strong statistical evidence for DFS and DRFS (Supplementary Figs. S11C and S12C). Its association with OS was presented as a visible trend by regression analyses, with a lack of statistical evidence (Supplementary Fig. S11F). This might correspond to the crossing of OS probability curves of the nonzero RCB classes observed at a later follow-up (Supplementary Fig. S11D).

### Association of sTILs with Prognosis

In univariable analysis without subtype stratification, we observed that a higher sTIL was associated with improved survival for all endpoints (Supplementary Figs. S13 and S14). Its role as an independent prognostic factor was not confirmed by multivariable analyses adjusting for other clinicopathologic and treatment variables, and pCR (Supplementary Figs. S13 and S14). Analyses according to receptor subgroups revealed a trend towards improved survival of patients with higher sTILs only in the ER^−^/HER2^+^ subgroup.

## Discussion

IBC represents a clinical breast cancer presentation which unfortunately is still under-investigated. In this study, we described, to the best of our knowledge, the largest retrospective cohort of primary IBC treated with neoadjuvant chemotherapy so far. We provide detailed characterization of clinicopathologic variables and central pathology review in relation to pCR, RCB, and survival outcomes.

Central pathology review illustrated the presence of tumor emboli in the skin for the 49.2% (30/61) patients for which pre-NACT skin biopsy was available. This is relatively lower than was described in the study by Hirko and colleagues (38); however, sampling before NACT of skin, for patients with IBC without direct skin invasion, is not standard in clinical practice, and was also not performed for a large majority of the patients in this cohort. For the majority of the patients only CNB were available and for some larger incisional biopsies were evaluable, which also might influence the findings of tumor emboli. This confirms again that the presence of tumor emboli on histology is not a prerequisite for the diagnosis of IBC (10), but still remains a remarkable phenomenon in IBC, and can be an aid when taken together with clinical presentation. The biological relevance of these emboli has already been suggested and should be further investigated (39–41).

sTILs in IBC were generally lower in comparison with what has been described in literature for IBC (sTILs were <10% in 64.6% in our cohort versus 35.8% in the cohort of Van Berckelaer and colleagues (26) and sTIL were <15% in 48.3% in the cohort of Arias-Pulido and colleagues (19)). In comparison with the general breast cancer population, sTILs were generally lower in our IBC cohort with sTIL < 10% in 64.6% of the cases versus 44% in general breast cancer population, as well as in the respective receptor subgroups (sTIL < 10% for 75.4% of ER^+^/HER2^−^; for 58.9% of HER2^+^ and 52.2% for ER^−^/HER2^−^ subgroup in our IBC cohort versus sTIL < 10% in 56% of Luminal breast cancer, 44% in HER2^+^ breast cancer, and 29% in TNBC for the general breast cancer population; ref. 28). Similar to the previous reported general breast cancer population, relatively higher sTILs were observed in the ER^−^ groups in comparison with the ER^+^ groups (19, 26, 28).

In our series, the overall pCR rate was 26.3% and is well within the range reported for IBC in the literature (9%–40%; refs. 18, 19, 25–27, 42). This is also comparable to the pCR rate reported by Denkert and colleagues but slightly lower than the 32.5% reported by Yau and colleagues in a general breast cancer population (20, 24, 28). In our series, we have a relatively higher proportion of patients with HER2^+^ tumors not receiving neoadjuvant anti-HER2 therapy (35.9% in our series vs. 13% in study by Yau and colleagues; ref. 24), and a numerical increase of pCR rate was observed in the group receiving anti-HER2 treatment, hereby likely underestimating pCR rates in our study in this subgroup of patients. However, studies comparing IBC with stage-matched non-IBC have shown variable results with either a remarkable difference in pCR (IBC 9% vs. non-IBC 20%; ref. 19) or similar pCR rates (11). In the latter study, survival was still impaired for patients with IBC, despite pCR (11). In our study, the addition of taxanes to the NACT was independently associated with increased pCR for all patients. Considering subgroups, the addition of taxane was only associated with pCR in the HER2^+^ subgroups, and especially for HER2^+^ patients not receiving anti-HER2 therapy. Our results therefore may challenge the recommended use of taxane-based NACT irrespective of subgroup consideration (10, 26, 43).

Similar to non-IBC, higher pCR rates for HER2^+^ and ER^−^ subgroups were observed, however, we observed an absolute lower pCR rate in the ER^−^/HER2^−^ subgroup in contrast to non-IBC (24, 28). Neoadjuvant anti-HER2 therapy was associated with higher pCR rates in unadjusted analysis, but not after adjusting for other clinicopathologic variables. This might be due to the additional effect of NACT, given together with anti-HER2 therapy. Prospective trials with neoadjuvant anti-HER2 including patients with IBC have been performed demonstrating improved pCR rates with the addition of pertuzumab to NACT-trastuzumab (44, 45), and improved outcomes with continuation of anti-HER2 treatment in the adjuvant setting (46); however, there was no formal reporting on the results specifically for the IBC cohort.

pCR was independently associated with HER2^+^ and ER^−^ status, as well as tumor grade 3, and receipt of taxane-based chemotherapy in the entire cohort. The association with HER2^+^ status is possibly mediated by the effect of anti-HER2 therapy. This was only given in 62.8% of patients with HER2^+^ IBC, although a numerically higher pCR rate in patients receiving neoadjuvant anti-HER2 therapy was observed. The effect of the clinicopathologic variables of interest beyond ER and HER2 varied across the different subgroups, supporting the fact that the biology and thus stratification according to receptor status remains important in IBC. Reaching pCR was independently associated with DFS and OS in the different subgroups except for OS in the ER^+^/HER2^−^ group where only a trend could be observed. ER negativity and HER2 negativity were each associated with poorer DFS and OS in multivariable analyses. This is in line with previously reported impaired outcomes of ER^−^ or HER2^−^ IBC in comparison with ER^+^ and HER2^+^ IBC (11, 18, 26, 47), but conflicts with the results presented by Liu and colleagues In that study, the authors reported that pCR is not prognostic and that ER^+^ IBC was not associated with better outcomes (42).

RCB classification was available for a subset of patients, in which we were able to show the prognostic dimension of RCB class. These results are in line with the results from the analyses of pCR and what has been reported for classic forms of breast cancer in general (24, 29). In patients with residual disease, a lower RCB score was associated with better DFS and DRFS. An association of lower RCB score with better OS was observed in early setting of follow-up of these patients, which was however diminished at a later follow-up time, that is, 10 years. Nevertheless, this needs to be interpreted with caution given the low numbers in this cohort.

Our results show that high sTIL are predictive for reaching pCR in the entire cohort; however, within the different receptor subgroups, this association could not be seen. This is in contrast to what has been described for the general breast cancer population where sTILs were predictive for pCR for all patients and for all breast cancer receptor subgroups (28). The prognostic effect of sTIL on OS is reduced when considering pCR in our series, which is similar to what has been described in the general breast cancer population (28), a trend could be observed for improved DFS and OS with increasing sTIL, even in the ER^+^/HER2^−^ subgroups.

It has been reported that the immune microenvironment is different in IBC from non-IBC, with relatively higher IHC expression of PD-L1 on the sTILs, especially on B lymphocytes, and that these differences may have a prognostic significance (19, 26, 48). Moreover, Bertucci and colleagues demonstrated higher expression of different immune checkpoint molecules in comparison to non-IBC such as *TIM3*, *CD27*, *CD70*, *CTLA4*, *ICOS*, *IDO1*, *LAG3*, *PDCD1*, *TNFRSF9*, *PVRIG*, *CD274* (PD-L1), and *TIGIT* (49). These data suggest that a relatively higher proportion of patients with IBC might be suitable candidates for immune checkpoint therapy (ICT). Patients in our study did not receive ICT, and in the past patients with IBC were often excluded from clinical trials because of the aggressive behavior of their disease. The PELICAN phase II trial (PELICAN-IPC 2015–016/Oncodistinct-003; NCT03515798), investigating the addition of pembrolizumab to standard NACT regimen for patients with HER2^−^ IBC, is trying to address this treatment inequality (50).

Our study presents three main limitations. First, given the selection criteria of this study, there might be a selection bias towards more aggressive subtypes of breast cancer because in the past and also still today, patients with ER^+^ IBC might have been more likely to be treated like ER^+^ non-IBC, not receiving NACT.

A second limitation of this study, and in general of retrospective studies on IBC, is its definition, which is currently based on a set of clinical symptoms. These are prone to subjective interpretation and are not always described in detail in the historical clinical files. Recent efforts by Jagsi and colleagues have elaborated the clinical criteria for IBC with a weighted scoring system and allows more standardization of the diagnosis (51). This will be validated prospectively and has the potential of homogenizing IBC diagnosis within and across hospitals.

No central pathology review was performed for ER and HER2 status, nevertheless the distribution of patients in the receptor subgroups in this study is similar to what has been reported before in IBC studies investigating pCR, demonstrating a relatively higher proportion of ER^−^/HER2^−^ and HER2^+^ tumors (18, 19, 25–27, 42). Results reported in the different receptor subgroups need to be observed with caution as numbers and events per subgroup were sometimes low.

Finally, in this study, we have not collected data concerning race, given the retrospective nature of this study and the difficulties of categorizing races. It has, however, been reported that IBC is overrepresented in black women and that there is an impaired prognosis for black women with IBC in comparison with non-black woman (1). As this is a European cohort, one needs to be cautious comparing U.S. and European cohorts due to possible demographic differences and (access to) healthcare systems.

In conclusion, this is the largest retrospective study so far for IBC with central pathology review. We have demonstrated that sTIL was predictive of pCR, although this could not be confirmed in stratified analyses by subgroups, and that pCR was associated with better prognosis. The independent prognostic value of sTILs could however not be demonstrated in this series. We have further confirmed the importance of stratification according to ER and HER2 status in IBC. Efforts need to be made to improve pCR rates and survival rates, especially for patients with ER^−^/HER2^−^ IBC.

## Supplementary Material

Supplementary Table 1 Supplementary Table 1 shows clinicopathological characteristics of the entire cohort and the sub-cohort with central pathology performed.

Supplementary Table 2 Supplementary Table 2 shows clinicopathological characteristics of the entire cohort and the Leuven sub-cohort.

Supplementary Table 3 Supplementary Table 3 shows pCR rates and sTIL according to histology.

Supplementary Figure 1 Supplementary Figure 1 shows examples of sTIL scoring in skin on H&E images.

Supplementary Figure 2 Supplementary Figure 2 shows an overview of data flow in the study.

Supplementary Figure 3 Supplementary Figure 3 shows concordance of sTIL scoring between tumor and skin biopsies.

Supplementary Figure 4 Supplementary Figure 4 shows an overview of tumor emboli assessment.

Supplementary Figure 5 Supplementary Figure 5 shows quantile regression analyses of sTIL with clinicopathological variables.

Supplementary Figure 6 Supplementary Figure 6 shows subgroup analyses of the association of sTIL with clinicopathological variables.

Supplementary Figure 7 Supplementary Figure 7 shows subgroup analyses of the association of pCR with clinicopathological and treatment variables.

Supplementary Figure 8 Supplementary Figure 8 shows descriptive data of pCR rates according to several pathological features.

Supplementary Figure 9 Supplementary Figure 9 shows potential non-linear association of RCB score with sTIL.

Supplementary Figure 10 Supplementary Figure 10 shows analyses of the association of pCR with DRFS.

Supplementary Figure 11 Supplementary Figure 11 shows analyses of the association of RCB with DRF and OS.

Supplementary Figure 12 Supplementary Figure 12 shows analyses of the association of RCB with DRFS.

Supplementary Figure 13 Supplementary Figure 13 shows analyses of the association of sTIL with DFS and OS.

Supplementary Figure 14 Supplementary Figure 14 shows analyses of the association of sTIL with DRFS.
