# Supplementary material for: Treatment Response, Tumor Infiltrating Lymphocytes and Clinical Outcomes in Inflammatory Breast Cancer–Treated with Neoadjuvant Systemic Therapy
Source: Cancer Res Commun. 2024 Jan 24;4(1):186–99. doi: 10.1158/2767-9764.CRC-23-0285 (PMC10807408; doi:10.1158/2767-9764.CRC-23-0285)
Supplement: Supplementary Table 1 — shows clinicopathological characteristics of the entire cohort and the sub-cohort with central pathology performed. [file crc-23-0285-s01.pdf]

Supplementary Table 1. Comparison of clinical and pathological characteristics of patients in the entire cohort and in the sub-cohort with central pathology performed

		All<br>N (%)	Central pathology<br>N (%)	p-value
**Age**	**≤ 50**	194 (39.3%)	135 (39.9)	0.885
	**> 50**	300 (60.7%)	203 (60.1)	
**Menopausal status**	**Pre/Peri-menopausal**	192 (41.8)	137 (42.8)	0.825
	**Post-menopausal**	267 (58.2)	183 (57.2)	
	**Unknown**	35	18	
**BMI category**	**Underweight**	3 (0.6)	2 (0.6%)	0.947
	**Lean**	165 (35.4)	113 (34.8)	
	**Overweight**	159 (34.1)	107 (32.9)	
	**Obese**	139 (29.8)	103 (31.7)	
	**Unknown**	28	13	
**Focality**	**Unifocal**	230 (79.6)	160 (76.9)	0.508
	**Multifocal**	59 (20.4)	48 (23.1)	
	**Unknown**	205	130	
**Histology**	**ILC**	30 (6.9)	16 (5.6)	0.767
	**NST**	400 (91.3)	265 (92.7)	
	**Other**	8 (1.8)	5 (1.7)	
	**Unknown**	56	52	
**Grade**	**G1**	15 (3.4)	10 (3.1)	0.909
	**G2**	141 (32.0)	106 (33.3)	
	**G3**	285 (64.6)	202 (63.5)	
	**Unknown**	53	20	
**ER status**	**Negative**	229 (48.5)	148 (45.0)	0.350
	**Positive**	243 (51.5)	181 (55.0)	
	**Unknown**	22	9	
**HER2 status**	**Negative**	297 (63.6)	207 (63.9)	0.940
	**Positive**	170 (36.4)	117 (36.1)	
	**Unknown**	27	14	
**PR status**	**Negative**	295 (64.6)	203 (63.2)	0.762
	**Positive**	162 (35.5)	118 (36.8)	
	**Unknown**	37	17	
**Lymph node positivity**	**No**	88 (18.5)	59 (17.7)	0.853
	**Yes**	389 (81.6)	275 (82.3)	
	**Unknown**	17	4	
**Neoadjuvant anti-HER2**	**No**	368 (76.7)	251 (74.9)	0.617
	**Yes**	112 (23.3)	84 (25.1)	
	**Unknown**	14	3	
**Neoadjuvant chemotherapy scheme**	**Taxane**	384 (79.7)	275 (82.1)	0.418
	**No Taxane**	98 (20.3)	60 (17.9)	
	**Unknown**	12	3	

**Surgery**	**Mastectomy**	367 (96.8)	244 (97.6)	0.634
	**Tumorectomy**	12 (3.2)	6 (2.4)	
	**Unknown**	115	88	
**Radiotherapy**	**No**	14 (3.3)	9 (3.2)	1.000
	**Yes**	412 (96.7)	274 (96.8)	
	**Unknown**	68	55	
**pCR**	**No pCR**	355 (73.7)	86 (25.9%)	0.935
	**pCR**	127 (26.4)	246 (74.1%)	
	**Unknown**	12	6	
**RCB class[#]**	**0**	40 (28.2)	27 (23.7)	0.801
	**I**	12 (8.4)	12 (10.5)	
	**II**	45 (31.7)	40 (35.1)	
	**III**	45 (31.7)	35 (30.7)	
	**Unknown**	21	7	

[#] Statistics are described for patients diagnosed and treated at the University Hospitals Leuven
