# Supplementary material for: Treatment Response, Tumor Infiltrating Lymphocytes and Clinical Outcomes in Inflammatory Breast Cancer–Treated with Neoadjuvant Systemic Therapy
Source: Cancer Res Commun. 2024 Jan 24;4(1):186–99. doi: 10.1158/2767-9764.CRC-23-0285 (PMC10807408; doi:10.1158/2767-9764.CRC-23-0285)
Supplement: Supplementary Table 2 — shows clinicopathological characteristics of the entire cohort and the Leuven sub-cohort. [file crc-23-0285-s02.pdf]

Supplementary Table 2. Comparison of clinical and pathological characteristics of patients in the entire cohort and patients in the Leuven cohort

		All<br>N (%)	Leuven cohort<br>N (%)	p-value
**Age**	**≤ 50**	194 (39.3%)	59 (36.2%)	0.517
	**> 50**	300 (60.7%)	104 (63.8%)	
**Menopausal status**	**Pre/Peri-menopausal**	192 (41.8)	71 (43.8)	0.712
	**Post-menopausal**	267 (58.2)	91 (56.2)	
	**Unknown**	35	1	
**BMI category**	**Underweight**	3 (0.6)	0 (0%)	0.557
	**Lean**	165 (35.4)	56 (34.4)	
	**Overweight**	159 (34.1)	64 (39.3)	
	**Obese**	139 (29.8)	43 (26.4)	
	**Unknown**	28	0	
**Focality**	**Unifocal**	230 (79.6)	143 (87.7)	0.029
	**Multifocal**	59 (20.4)	20 (12.3)	
	**Unknown**	205	0	
**Histology**	**ILC**	30 (6.9)	15 (9.4)	0.334
	**NST**	400 (91.3)	140 (87.5)	
	**Other**	8 (1.8)	5 (3.1)	
	**Unknown**	56	3	
**Grade**	**G1**	15 (3.4)	5 (3.1)	0.730
	**G2**	141 (32.0)	57 (35.4)	
	**G3**	285 (64.6)	99 (61.5)	
	**Unknown**	53	2	
**ER status**	**Negative**	229 (48.5)	74 (47.1)	0.782
	**Positive**	243 (51.5)	83 (52.9)	
	**Unknown**	22	6	
**HER2 status**	**Negative**	297 (63.6)	84 (56.4)	0.122
	**Positive**	170 (36.4)	65 (43.6)	
	**Unknown**	27	14	
**PR status**	**Negative**	295 (64.6)	91 (58.3)	0.179
	**Positive**	162 (35.5)	65 (41.7)	
	**Unknown**	37	7	
**Lymph node positivity**	**No**	88 (18.5)	20 (12.3)	0.070
	**Yes**	389 (81.6)	143 (87.7)	
	**Unknown**	17	0	
**Neoadjuvant anti-HER2**	**No**	368 (76.7)	115 (70.5)	0.142
	**Yes**	112 (23.3)	48 (29.5)	
	**Unknown**	14	0	
**Neoadjuvant chemotherapy scheme**	**Taxane**	384 (79.7)	146 (89.6)	0.004
	**No Taxane**	98 (20.3)	17 (10.4)	
	**Unknown**	12	0	

**Surgery**	**Mastectomy**	367 (96.8)	155 (96.3)	0.794
	**Tumorectomy**	12 (3.2)	6 (3.7)	
	**Unknown**	115	2	
**Radiotherapy**	**No**	14 (3.3)	2 (1.2)	0.257
	**Yes**	412 (96.7)	161 (98.8)	
	**Unknown**	68	0	
**pCR**	**No pCR**	355 (73.7)	123 (75.5)	0.680
	**pCR**	127 (26.4)	40 (24.5)	
	**Unknown**	12	0	
