# Supplementary material for: Treatment Response, Tumor Infiltrating Lymphocytes and Clinical Outcomes in Inflammatory Breast Cancer–Treated with Neoadjuvant Systemic Therapy
Source: Cancer Res Commun. 2024 Jan 24;4(1):186–99. doi: 10.1158/2767-9764.CRC-23-0285 (PMC10807408; doi:10.1158/2767-9764.CRC-23-0285)
Supplement: Supplementary Table 3 — shows pCR rates and sTIL according to histology. [file crc-23-0285-s03.pdf]

## Supplementary Table 3. pCR rates and sTIL according to tumor histology in different surrogate molecular subgroups

| | | ER-/HER2- | | | | ER-/HER2+ | | | | ER+/HER2- | | | | ER+/HER2+ | | | |
|---|---|---|---|---|---|---|---|---|---|---|---|---|---|---|---|---|---|
| | | NST | ILC | Others | p-value | NST | ILC | Others | p-value | NST | ILC | Others | p-value | NST | ILC | Others | p-value |
| pCR | No | 66 (73.3%) | 5 (83.3%) | 1 (100%) | 1.000 | 45 (54.2%) | 1 (50.0%) | 0 (0.0%) | 0.735 | 113 (87.6%) | 14 (100%) | 5 (100%) | 0.425 | 30 (53.6) | 4 (80.0) | 1 (100.0) | 0.472 |
| | Yes | 24 (26.7%) | 1 (16.7%) | 0 (0.0%) | | 38 (45.8%) | 1 (50.0%) | 1 (100%) | | 16 (12.4%) | 0 (0.0%) | 0 (0.0%) | | 26 (46.4) | 1 (20.0) | 0 (0.0) | |
| sTIL (%) | Median (IQR) | 10.0 [5.0; 20.0] | 3.1 [2.7; 3.5] | NA | 0.140 | 8.5 [3.3; 17.1] | 2.3 [2.3; 2.3] | 13.3 [13.3; 13.3] | 0.520 | 3.33 [0.67; 11.17] | 2.00 [0.67; 2.33] | 0.33 [0.33; 3.67] | 0.140 | 6.7 [3.3; 18.3] | 2.3 [1.2; 5.3] | 5.0 [5.0; 5.0] | 0.390 |
| sTIL (category) | Low | 25 (51.0%) | 2 (100%) | 0 | 0.492 | 28 (53.8%) | 1 (100%) | 0 (0.0%) | 0.716 | 67 (72.8%) | 9 (100%) | 3 (100%) | 0.123 | 23 (62.2%) | 3 (100%) | 1 (100%) | 0.702 |
| | Intermediate/ High | 24 (49.0%) | 0 (0.0%) | 0 | | 24 (46.2%) | 0 (0.0%) | 1 (100%) | | 25 (27.2%) | 0 (0.0%) | 0 (0.0%) | | 14 (37.8%) | 0 (0.0%) | 0 (0.0%) | |
