# Supplementary material for: Treatment Response, Tumor Infiltrating Lymphocytes and Clinical Outcomes in Inflammatory Breast Cancer–Treated with Neoadjuvant Systemic Therapy
Source: Cancer Res Commun. 2024 Jan 24;4(1):186–99. doi: 10.1158/2767-9764.CRC-23-0285 (PMC10807408; doi:10.1158/2767-9764.CRC-23-0285)
Supplement: Supplementary Figure 1 — shows examples of sTIL scoring in skin on H&E images. [file crc-23-0285-s04.pdf]

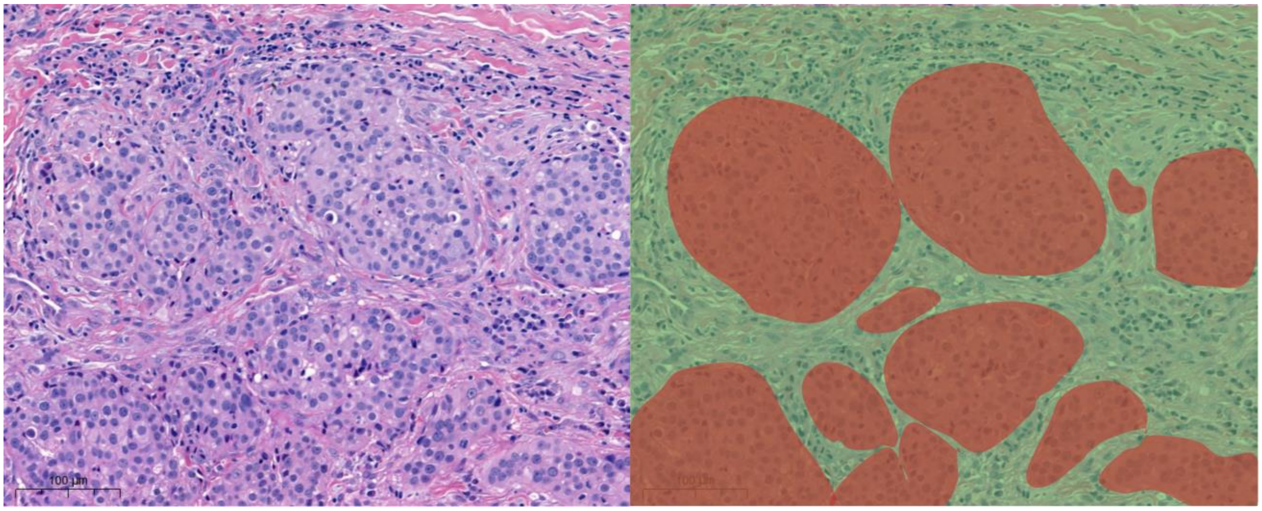

Supplementary Figure 1. sTIL scoring in skin samples. On the left an H&E image of invasive carcinoma in the dermis is depicted. On the right is the same H&E with green color overlay depicting the are in which sTIL are scored. The red transparent areas represent the tumor nests that are excluded from sTIL scoring. Images were taken at 40x, for illustrative purposes. sTIL were scored at a magnification of 20.
