# Supplementary material for: Treatment Response, Tumor Infiltrating Lymphocytes and Clinical Outcomes in Inflammatory Breast Cancer–Treated with Neoadjuvant Systemic Therapy
Source: Cancer Res Commun. 2024 Jan 24;4(1):186–99. doi: 10.1158/2767-9764.CRC-23-0285 (PMC10807408; doi:10.1158/2767-9764.CRC-23-0285)
Supplement: Supplementary Figure 2 — shows an overview of data flow in the study. [file crc-23-0285-s05.pdf]

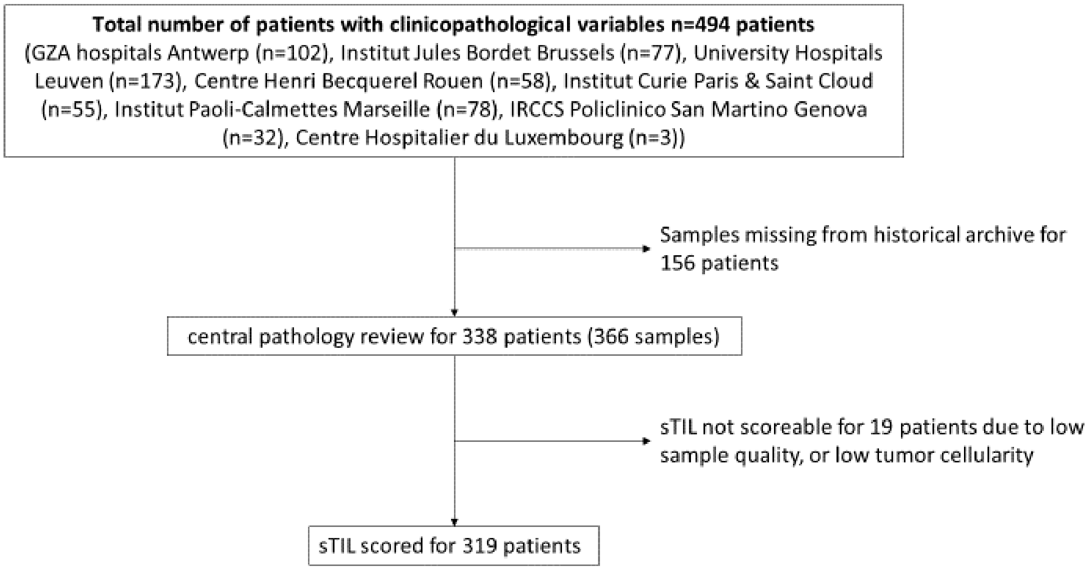

Supplementary Figure 2. Overview of the data flow. Flowchart illustrating number of patients through the processes of sample retrieval, quality control and central pathology review.
