# Supplementary material for: Treatment Response, Tumor Infiltrating Lymphocytes and Clinical Outcomes in Inflammatory Breast Cancer–Treated with Neoadjuvant Systemic Therapy
Source: Cancer Res Commun. 2024 Jan 24;4(1):186–99. doi: 10.1158/2767-9764.CRC-23-0285 (PMC10807408; doi:10.1158/2767-9764.CRC-23-0285)
Supplement: Supplementary Figure 3 — shows concordance of sTIL scoring between tumor and skin biopsies. [file crc-23-0285-s06.pdf]

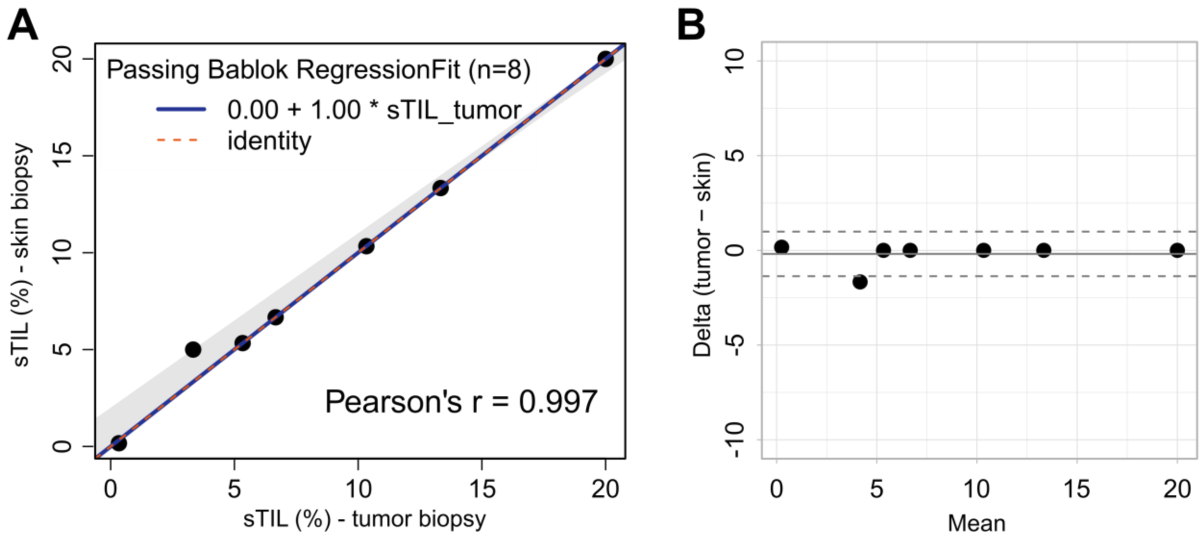

Supplementary Figure 3. Concordance analysis (tumor vs skin) of sTIL. (A) Passing-Bablok regression of sTIL scoring in tumor biopsies versus in skin biopsies; (B) Bland-Altman plot comparing sTIL in tumor biopsies and in skin biopsies.
