# Supplementary material for: Treatment Response, Tumor Infiltrating Lymphocytes and Clinical Outcomes in Inflammatory Breast Cancer–Treated with Neoadjuvant Systemic Therapy
Source: Cancer Res Commun. 2024 Jan 24;4(1):186–99. doi: 10.1158/2767-9764.CRC-23-0285 (PMC10807408; doi:10.1158/2767-9764.CRC-23-0285)
Supplement: Supplementary Figure 4 — shows an overview of tumor emboli assessment. [file crc-23-0285-s07.pdf]

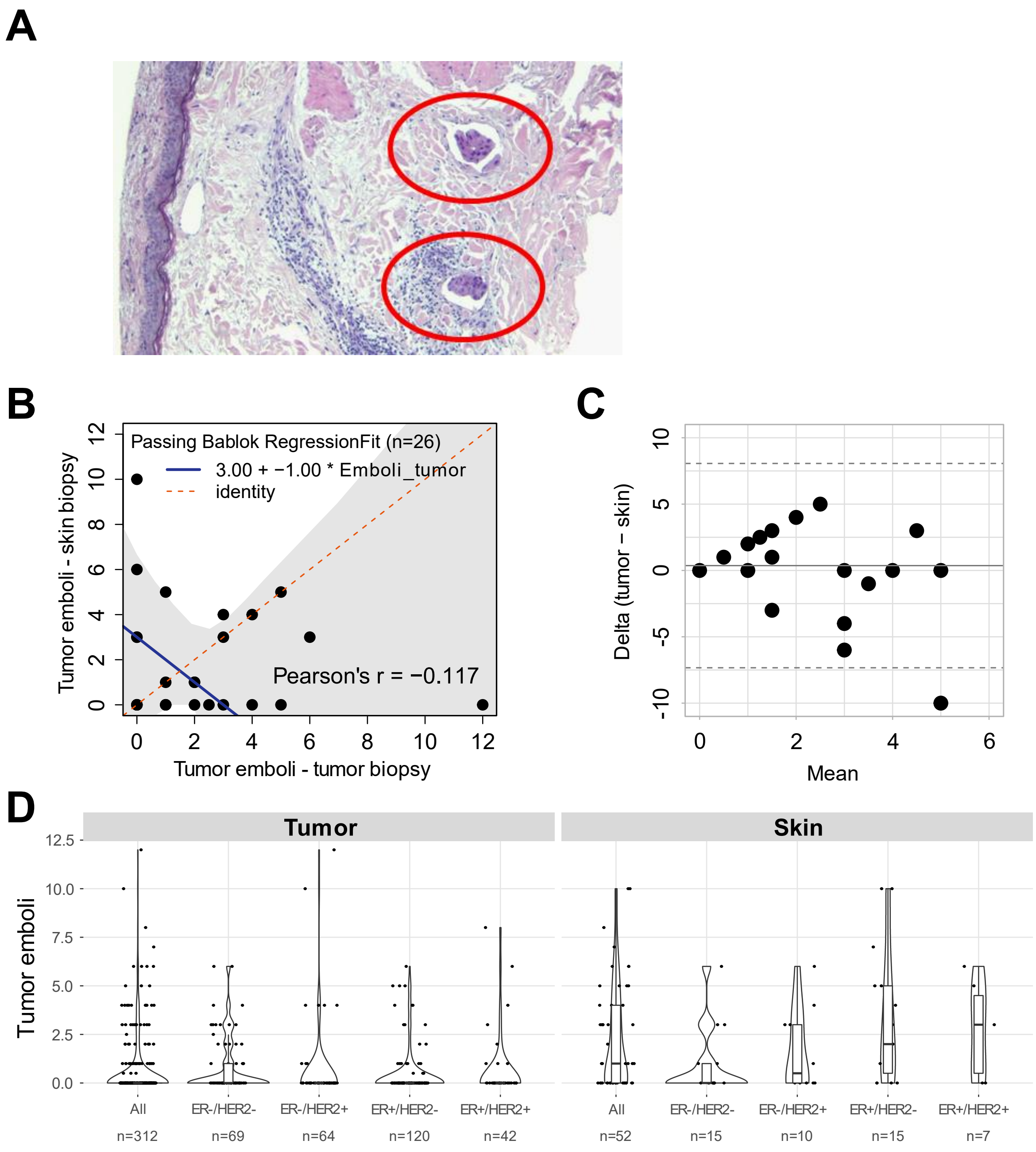

Supplementary Figure 4. Histopathological assessment of tumor emboli. (A) Skin biopsy illustrating presence of tumor emboli with the dermal lymphatics (red circles); (B) Passing-Bablok regression of tumor emboli in tumor biopsies versus in skin biopsies; (C) Bland-Altman plot comparing tumor emboli in tumor biopsies and in skin biopsies; (D) Distribution of tumor emboli by subtype.
