# Supplementary material for: Treatment Response, Tumor Infiltrating Lymphocytes and Clinical Outcomes in Inflammatory Breast Cancer–Treated with Neoadjuvant Systemic Therapy
Source: Cancer Res Commun. 2024 Jan 24;4(1):186–99. doi: 10.1158/2767-9764.CRC-23-0285 (PMC10807408; doi:10.1158/2767-9764.CRC-23-0285)
Supplement: Supplementary Figure 5 — shows quantile regression analyses of sTIL with clinicopathological variables. [file crc-23-0285-s08.pdf]

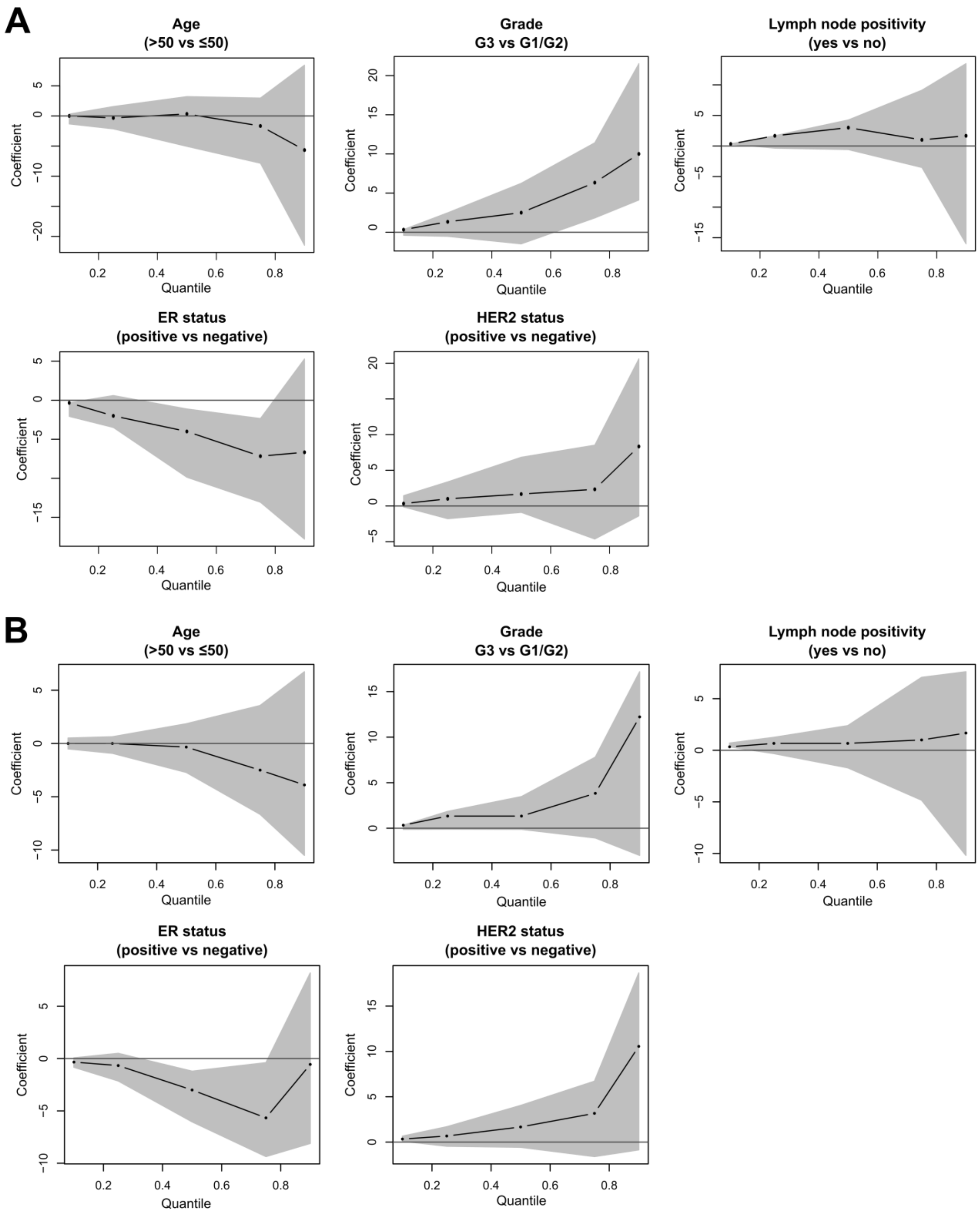

Supplementary Figure 5. Quantile regressions of sTIL with standard clinicopathological variables in all patients. (A) Plots displaying coefficients and their confidence intervals estimated at different quantiles (0.1-, 0.25-, 0.5-, 0.75-, and 0.9-quantiles) of sTIL (continuous %) for each of the clinicopathological variables of interest in a univariable model (A) and a multivariable model (B).
