# Supplementary material for: Treatment Response, Tumor Infiltrating Lymphocytes and Clinical Outcomes in Inflammatory Breast Cancer–Treated with Neoadjuvant Systemic Therapy
Source: Cancer Res Commun. 2024 Jan 24;4(1):186–99. doi: 10.1158/2767-9764.CRC-23-0285 (PMC10807408; doi:10.1158/2767-9764.CRC-23-0285)
Supplement: Supplementary Figure 6 — shows subgroup analyses of the association of sTIL with clinicopathological variables. [file crc-23-0285-s09.pdf]

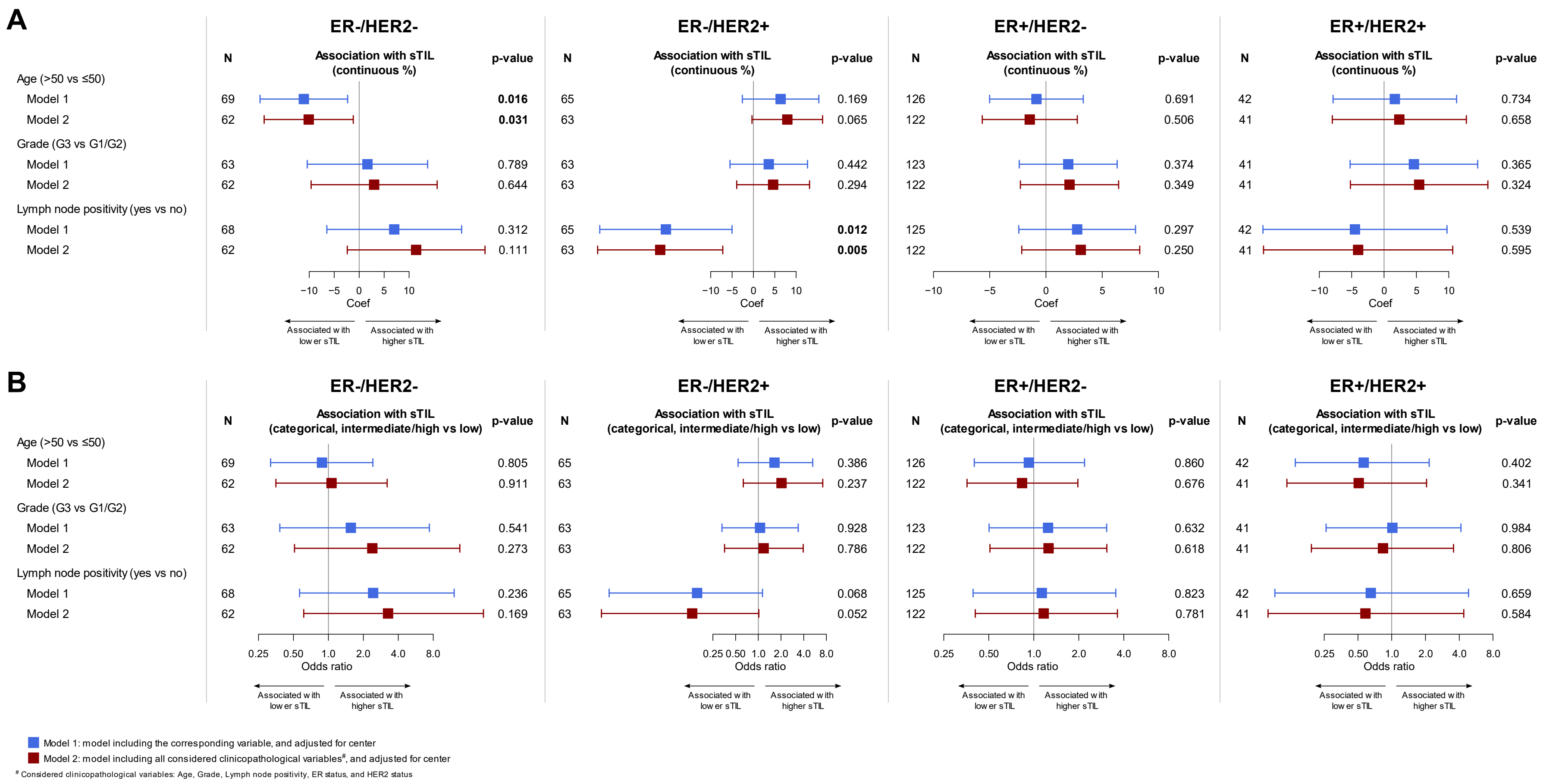

Supplementary Figure 6. Association of sTIL with clinicopathological variables in surrogate molecular subgroups. (A-B) Forest plots showing the association of sTIL (continuous) (A) and sTIL (categorical) (B) with standard clinicopathological variables evaluated by regression analyses in each subgroup.
