# Supplementary material for: Treatment Response, Tumor Infiltrating Lymphocytes and Clinical Outcomes in Inflammatory Breast Cancer–Treated with Neoadjuvant Systemic Therapy
Source: Cancer Res Commun. 2024 Jan 24;4(1):186–99. doi: 10.1158/2767-9764.CRC-23-0285 (PMC10807408; doi:10.1158/2767-9764.CRC-23-0285)
Supplement: Supplementary Figure 7 — shows subgroup analyses of the association of pCR with clinicopathological and treatment variables. [file crc-23-0285-s10.pdf]

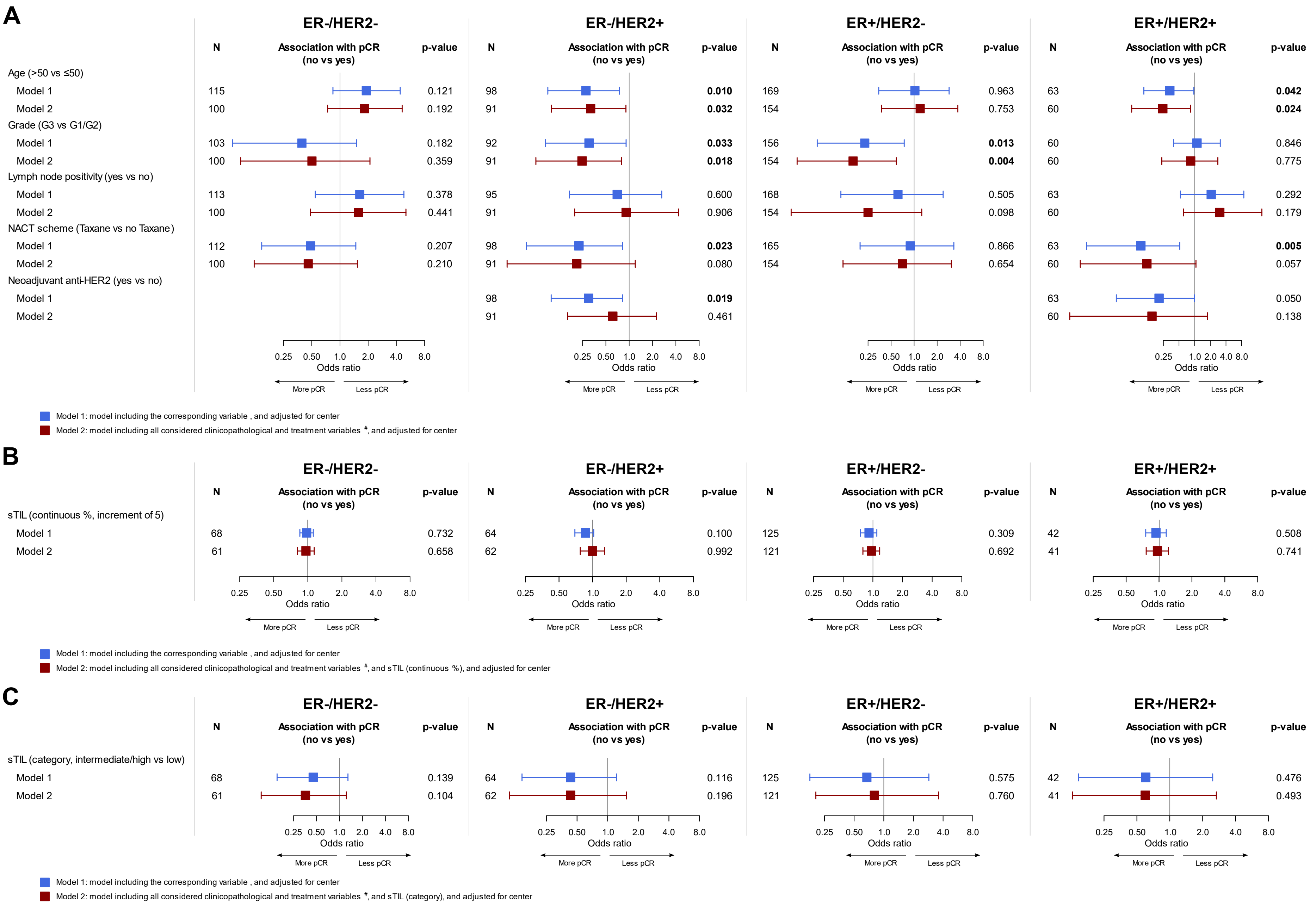

Considered clinicopathological and treatment variables: Age, Grade, Lymph node positivity, ER status, HER2 status, NACT scheme, and Neoadjuvant anti-HER2

Supplementary Figure 7. Association of pCR with clinicopathological features and treatment in surrogate molecular subgroups. (A-C) Forest plots showing the association of pCR with standard clinicopathological and treatment variables (A), with sTIL (continuous %) (B), and with sTIL (categorical) (C) evaluated by regression analyses in each subgroup.
