# Supplementary material for: Treatment Response, Tumor Infiltrating Lymphocytes and Clinical Outcomes in Inflammatory Breast Cancer–Treated with Neoadjuvant Systemic Therapy
Source: Cancer Res Commun. 2024 Jan 24;4(1):186–99. doi: 10.1158/2767-9764.CRC-23-0285 (PMC10807408; doi:10.1158/2767-9764.CRC-23-0285)
Supplement: Supplementary Figure 8 — shows descriptive data of pCR rates according to several pathological features. [file crc-23-0285-s11.pdf]

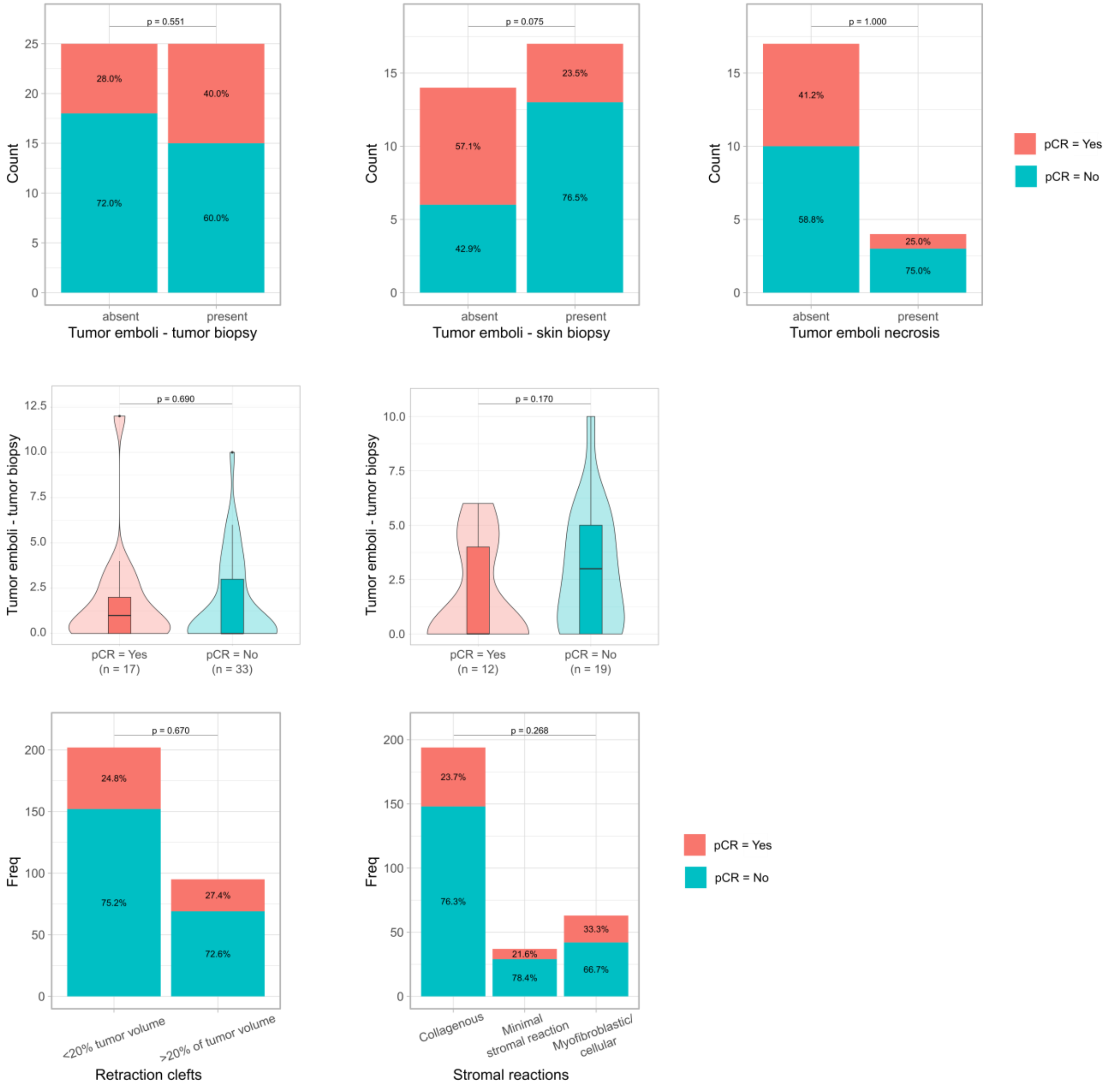

Supplementary Figure 8. The pCR rates according to other pathological features. Features explored include tumor emboli in tumor biopsies, tumor emboli in skin biopsies, tumor emboli necrosis, retraction lefts, and stromal reactions. Tumor emboli was considered either as a categorical variable (absent or present), or a continuous variable (density score).
