# Supplementary material for: Treatment Response, Tumor Infiltrating Lymphocytes and Clinical Outcomes in Inflammatory Breast Cancer–Treated with Neoadjuvant Systemic Therapy
Source: Cancer Res Commun. 2024 Jan 24;4(1):186–99. doi: 10.1158/2767-9764.CRC-23-0285 (PMC10807408; doi:10.1158/2767-9764.CRC-23-0285)
Supplement: Supplementary Figure 9 — shows potential non-linear association of RCB score with sTIL. [file crc-23-0285-s12.pdf]

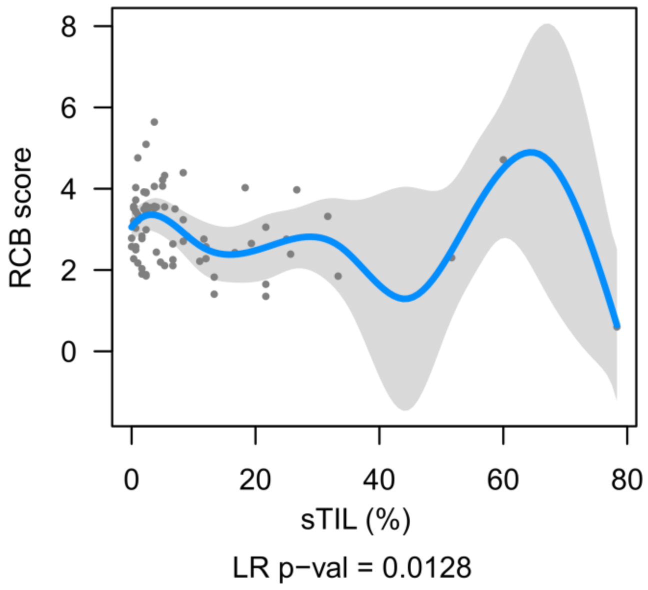

Supplementary Figure 9. Potential non-linear association of RCB score with sTIL in patients with residual disease. Plots showing fitted lines of the RCB score estimates, derived from the multivariable linear regression model fitted with a restricted cubic spline, against the values of sTIL scoring.

LR, likelihood ratio test
