# Supplementary material for: Treatment Response, Tumor Infiltrating Lymphocytes and Clinical Outcomes in Inflammatory Breast Cancer–Treated with Neoadjuvant Systemic Therapy
Source: Cancer Res Commun. 2024 Jan 24;4(1):186–99. doi: 10.1158/2767-9764.CRC-23-0285 (PMC10807408; doi:10.1158/2767-9764.CRC-23-0285)
Supplement: Supplementary Figure 11 — shows analyses of the association of RCB with DRF and OS. [file crc-23-0285-s14.pdf]

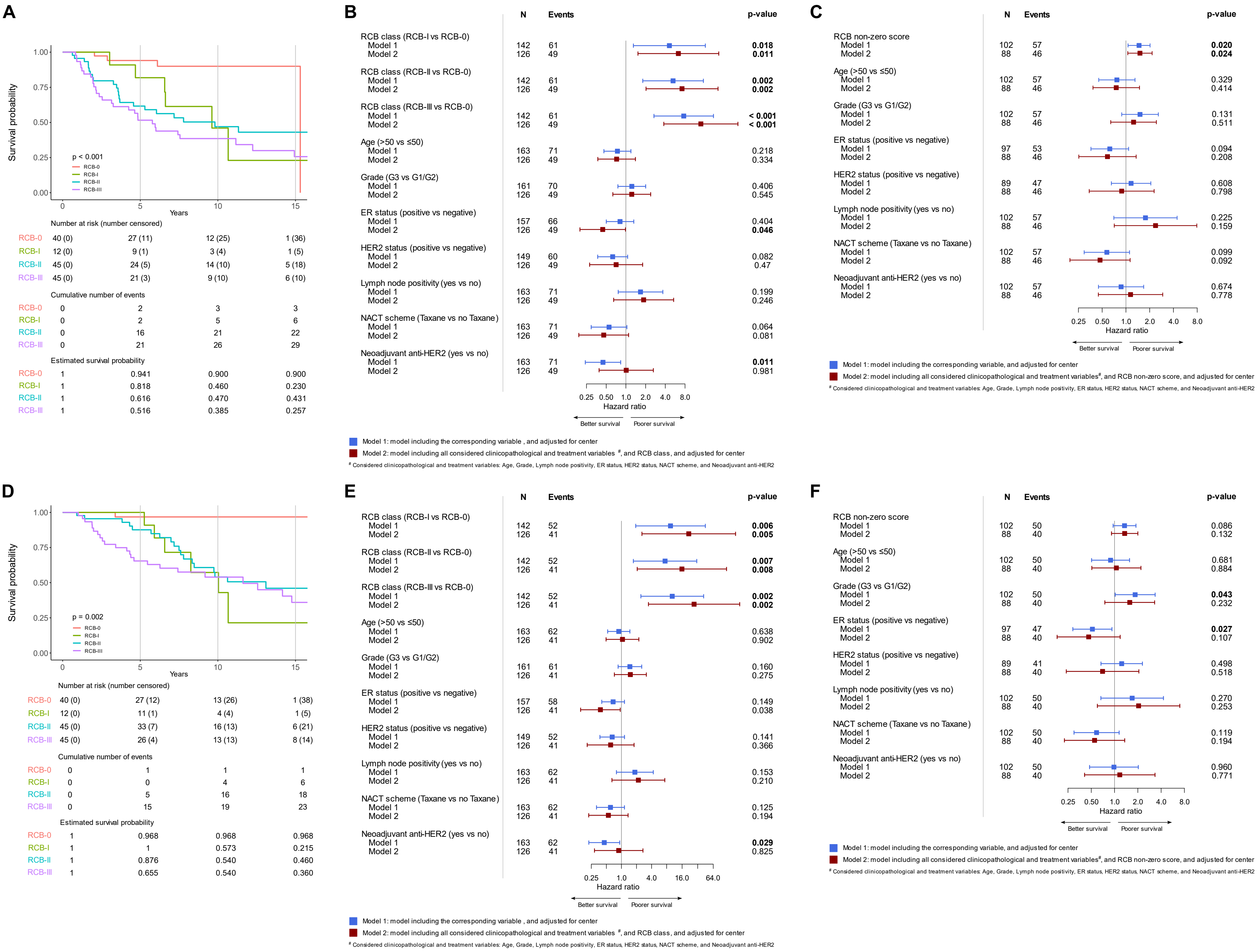
A
Survival probability
1.00
0.75
0.50
0.25
0.00
p < 0.001
RCB-0
RCB-I
RCB-II
RCB-III
0
5
10
15
Years
Number at risk (number censored)
RCB-0 40 (0) 27 (11) 12 (25) 1 (36)
RCB-I 12 (0) 9 (1) 3 (4) 1 (5)
RCB-II 45 (0) 24 (5) 14 (10) 5 (18)
RCB-III 45 (0) 21 (3) 9 (10) 6 (10)
Cumulative number of events
RCB-0 0 2 3 3
RCB-I 0 2 5 6
RCB-II 0 16 21 22
RCB-III 0 21 26 29
Estimated survival probability
RCB-0 1 0.941 0.900 0.900
RCB-I 1 0.818 0.460 0.230
RCB-II 1 0.616 0.470 0.431
RCB-III 1 0.516 0.385 0.257
B
N
Events
p-value
RCB class (RCB-I vs RCB-0)
Model 1 142 61 0.018
Model 2 126 49 0.011
RCB class (RCB-II vs RCB-0)
Model 1 142 61 0.002
Model 2 126 49 0.002
RCB class (RCB-III vs RCB-0)
Model 1 142 61 < 0.001
Model 2 126 49 < 0.001
Age (>50 vs ≤50)
Model 1 163 71 0.218
Model 2 126 49 0.334
Grade (G3 vs G1/G2)
Model 1 161 70 0.406
Model 2 126 49 0.545
ER status (positive vs negative)
Model 1 157 66 0.404
Model 2 126 49 0.046
HER2 status (positive vs negative)
Model 1 149 60 0.082
Model 2 126 49 0.47
Lymph node positivity (yes vs no)
Model 1 163 71 0.199
Model 2 126 49 0.246
NACT scheme (Taxane vs no Taxane)
Model 1 163 71 0.064
Model 2 126 49 0.081
Neoadjuvant anti-HER2 (yes vs no)
Model 1 163 71 0.011
Model 2 126 49 0.981
0.25 0.50 1.0 2.0 4.0 8.0
Hazard ratio
Better survival
Poorer survival
Model 1: model including the corresponding variable , and adjusted for center
Model 2: model including all considered clinicopathological and treatment variables #, and RCB class, and adjusted for center
Considered clinicopathological and treatment variables: Age, Grade, Lymph node positivity, ER status, HER2 status, NACT scheme, and Neoadjuvant anti-HER2
C
N
Events
p-value
RCB non-zero score
Model 1 102 57 0.020
Model 2 88 46 0.024
Age (>50 vs ≤50)
Model 1 102 57 0.329
Model 2 88 46 0.414
Grade (G3 vs G1/G2)
Model 1 102 57 0.131
Model 2 88 46 0.511
ER status (positive vs negative)
Model 1 97 53 0.094
Model 2 88 46 0.208
HER2 status (positive vs negative)
Model 1 89 47 0.608
Model 2 88 46 0.798
Lymph node positivity (yes vs no)
Model 1 102 57 0.225
Model 2 88 46 0.159
NACT scheme (Taxane vs no Taxane)
Model 1 102 57 0.099
Model 2 88 46 0.092
Neoadjuvant anti-HER2 (yes vs no)
Model 1 102 57 0.674
Model 2 88 46 0.778
0.25 0.50 1.0 2.0 4.0 8.0
Hazard ratio
Better survival
Poorer survival
Model 1: model including the corresponding variable, and adjusted for center
Model 2: model including all considered clinicopathological and treatment variables#, and RCB non-zero score, and adjusted for center
Considered clinicopathological and treatment variables: Age, Grade, Lymph node positivity, ER status, HER2 status, NACT scheme, and Neoadjuvant anti-HER2
D
Survival probability
1.00
0.75
0.50
0.25
0.00
p = 0.002
RCB-0
RCB-I
RCB-II
RCB-III
0
5
10
15
Years
Number at risk (number censored)
RCB-0 40 (0) 27 (12) 13 (26) 1 (38)
RCB-I 12 (0) 11 (1) 4 (4) 1 (5)
RCB-II 45 (0) 33 (7) 16 (13) 6 (21)
RCB-III 45 (0) 26 (4) 13 (13) 8 (14)
Cumulative number of events
RCB-0 0 1 1 1
RCB-I 0 0 4 6
RCB-II 0 5 16 18
RCB-III 0 15 19 23
Estimated survival probability
RCB-0 1 0.968 0.968 0.968
RCB-I 1 1 0.573 0.215
RCB-II 1 0.876 0.540 0.460
RCB-III 1 0.655 0.540 0.360
E
N
Events
p-value
RCB class (RCB-I vs RCB-0)
Model 1 142 52 0.006
Model 2 126 41 0.005
RCB class (RCB-II vs RCB-0)
Model 1 142 52 0.007
Model 2 126 41 0.008
RCB class (RCB-III vs RCB-0)
Model 1 142 52 0.002
Model 2 126 41 0.002
Age (>50 vs ≤50)
Model 1 163 62 0.638
Model 2 126 41 0.902
Grade (G3 vs G1/G2)
Model 1 161 61 0.160
Model 2 126 41 0.275
ER status (positive vs negative)
Model 1 157 58 0.149
Model 2 126 41 0.038
HER2 status (positive vs negative)
Model 1 149 52 0.141
Model 2 126 41 0.366
Lymph node positivity (yes vs no)
Model 1 163 62 0.153
Model 2 126 41 0.210
NACT scheme (Taxane vs no Taxane)
Model 1 163 62 0.125
Model 2 126 41 0.194
Neoadjuvant anti-HER2 (yes vs no)
Model 1 163 62 0.029
Model 2 126 41 0.825
0.25 1.0 4.0 16.0 64.0
Hazard ratio
Better survival
Poorer survival
Model 1: model including the corresponding variable , and adjusted for center
Model 2: model including all considered clinicopathological and treatment variables #, and RCB class, and adjusted for center
Considered clinicopathological and treatment variables: Age, Grade, Lymph node positivity, ER status, HER2 status, NACT scheme, and Neoadjuvant anti-HER2
F
N
Events
p-value
RCB non-zero score
Model 1 102 50 0.086
Model 2 88 40 0.132
Age (>50 vs ≤50)
Model 1 102 50 0.681
Model 2 88 40 0.884
Grade (G3 vs G1/G2)
Model 1 102 50 0.043
Model 2 88 40 0.232
ER status (positive vs negative)
Model 1 97 47 0.027
Model 2 88 40 0.107
HER2 status (positive vs negative)
Model 1 89 41 0.498
Model 2 88 40 0.518
Lymph node positivity (yes vs no)
Model 1 102 50 0.270
Model 2 88 40 0.253
NACT scheme (Taxane vs no Taxane)
Model 1 102 50 0.119
Model 2 88 40 0.194
Neoadjuvant anti-HER2 (yes vs no)
Model 1 102 50 0.960
Model 2 88 40 0.771
0.25 0.50 1.0 2.0 4.0 8.0
Hazard ratio
Better survival
Poorer survival
Model 1: model including the corresponding variable, and adjusted for center
Model 2: model including all considered clinicopathological and treatment variables#, and RCB non-zero score, and adjusted for center
Considered clinicopathological and treatment variables: Age, Grade, Lymph node positivity, ER status, HER2 status, NACT scheme, and Neoadjuvant anti-HER2

Supplementary Figure 11. Association of RCB with DFS and OS. (A) Kaplan-Meier curves of DFS according to RCB class; (B-C) Forest plots showing the association of RCB class (B), or non-zero RCB score (C), and standard clinicopathological and treatment variables with DFS quantified by Cox regression. (D) Kaplan-Meier curves of OS according to RCB class; (E-F) Forest plots showing the association of RCB class (E), or non-zero RCB score (F), and standard clinicopathological and treatment variables with OS quantified by Cox regression.
