# Supplementary material for: Treatment Response, Tumor Infiltrating Lymphocytes and Clinical Outcomes in Inflammatory Breast Cancer–Treated with Neoadjuvant Systemic Therapy
Source: Cancer Res Commun. 2024 Jan 24;4(1):186–99. doi: 10.1158/2767-9764.CRC-23-0285 (PMC10807408; doi:10.1158/2767-9764.CRC-23-0285)
Supplement: Supplementary Figure 12 — shows analyses of the association of RCB with DRFS. [file crc-23-0285-s15.pdf]

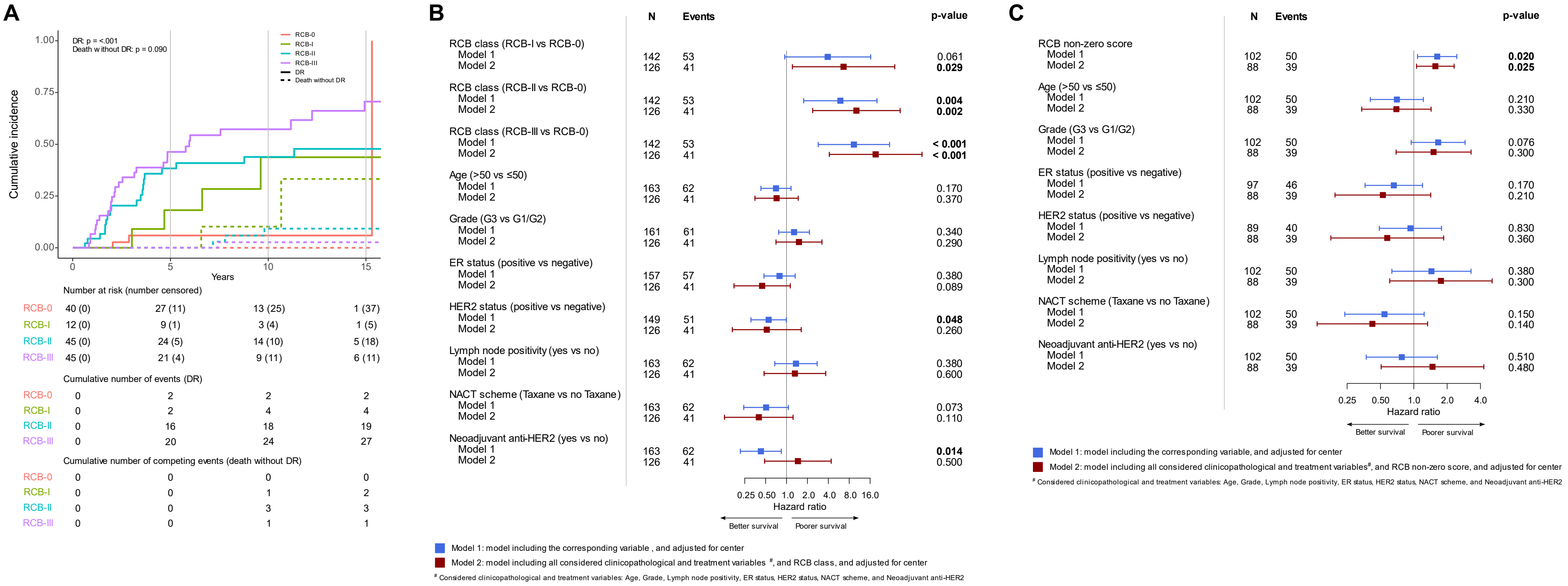

Supplementary Figure 12. Association of RCB with DRFS. (A) Cumulative incidence curves of distant recurrence (DR) and death without DR according to RCB class; (B-C) Forest plots showing the association of RCB class (B), or non-zero RCB score (C), and standard clinicopathological and treatment variables with DRFS quantified by Fine-Gray regression.
