# Supplementary material for: Treatment Response, Tumor Infiltrating Lymphocytes and Clinical Outcomes in Inflammatory Breast Cancer–Treated with Neoadjuvant Systemic Therapy
Source: Cancer Res Commun. 2024 Jan 24;4(1):186–99. doi: 10.1158/2767-9764.CRC-23-0285 (PMC10807408; doi:10.1158/2767-9764.CRC-23-0285)
Supplement: Supplementary Figure 13 — shows analyses of the association of sTIL with DFS and OS. [file crc-23-0285-s16.pdf]

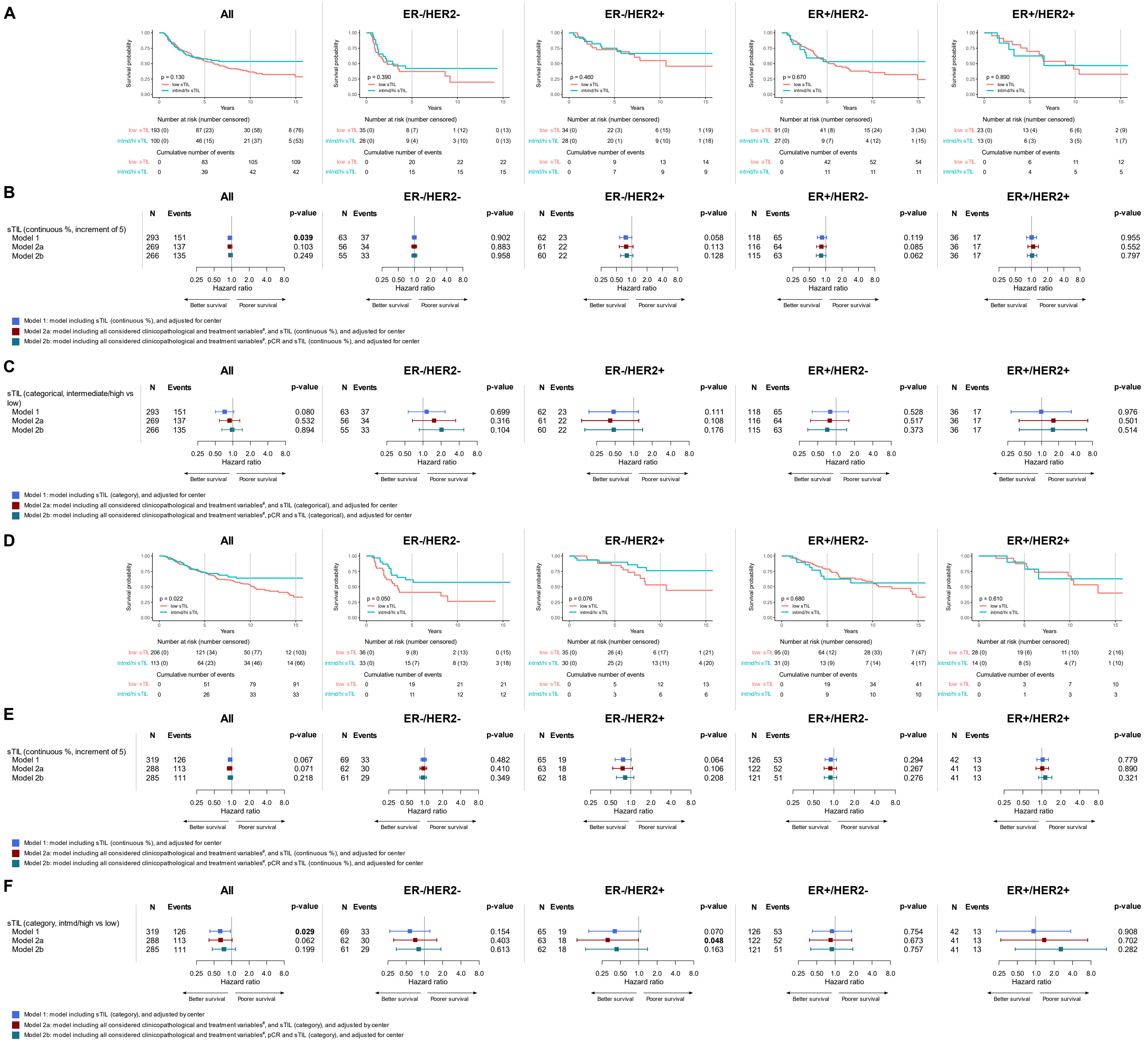

Considered clinicopathological and treatment variables: Age, Grade, Lymph node positivity, ER status, HER2 status, NACT scheme, and Neoadjuvant anti-HER2

Supplementary Figure 13. Association of sTIL with DFS and OS. (A) Kaplan-Meier curves of DFS according to sTIL category; (B-C) Forest plots showing the association of sTIL category (B), or sTIL (continuous %) (C) with DFS quantified by Cox regression. (D) Kaplan-Meier curves of OS according to sTIL category; (E-F) Forest plots showing the association of sTIL category (E), or sTIL (continuous %) (F) with OS quantified by Cox regression.
